# Well-defined benzoxazine/triphenylamine-based hyperbranched polymers with controlled degree of branching†

**DOI:** 10.1039/c8ra00506k

**Published:** 2018-04-11

**Authors:** Ruey-Chorng Lin, Shiao-Wei Kuo

**Affiliations:** Department of Materials and Optoelectronic Science, National Sun Yat-Sen University Kaohsiung 80424 Taiwan kuosw@faculty.nsysu.edu.tw

## Abstract

Well-defined thermally polymerizable hyperbranched polymers (TPA–BZs) containing various numbers of triphenylamine (TPA) and benzoxazine (BZ) units have been prepared using a “click-like” reaction concept, through one-pot Mannich condensations of 4-(bis(4-aminophenyl)amino)phenol (TPA–2NH_2_–OH, as the AB_2_ branching groups), aniline (as the focal groups), CH_2_O, and phenol in 1,4-dioxane, with a unique feeding approach. Two design strategies for the chemical construction were applied: (i) simple hyperbranched TPA–BZs, such as those containing one or three TPA units, developed from the focal or the terminal group direction to form the resultant monomers; (ii) three dendritic TPA–BZs containing four TPA units possessing different degrees of branching (DBs) for the conformation study. The exothermic temperature for the dendritic TPA–BZs decreased upon increasing the DB. The bathochromic shifts of the dendritic TPA–BZs increased upon increasing the number of TPA units, in UV-Vis absorption and PL emission spectra, presumably because of an increase in the effective conjugation length. In addition, the polymerized dendritic TPA–BZ DG1 possessed thermal properties superior to those of the hyperbranched TPA–BZ polybenzoxazines, possibly because the segmental mobility in the polymer network was restricted by the dendrimer core group and because of its symmetrical construction. The hyperbranched TPA–BZ possessed unique photophysical properties, suggesting potential applications in optoelectronic devices.

## Abstract 2

Different well-defined benzoxazine/triphenylamine based hyperbranched polymers with controlled degree of branching were prepared and discussed.

## Graphical abstract

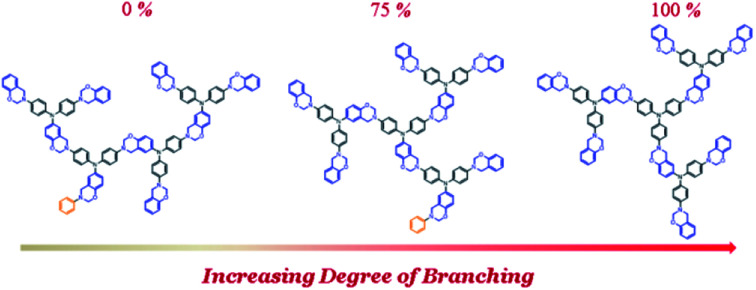

## Introduction

Hyperbranched polymers are asymmetrical counterparts to symmetrical dendrimers, but possess many of the unique features of dendrimers, including low viscosity, good solubility, terminal group functionality, and high branching density.^1,2^ In contrast to perfect dendrimers with uniform chemical constructions, the merits of imperfect hyperbranched polymers are often underestimated because they have some unattractive features—for example, uncontrolled molecular weight, broad distribution, and diversity in their chemical construction; in some cases they considered only the results as side reactions in polymerizations or as network formation precursors of dendrimers.^3^ The applications of dendrimers are, however, restricted by their high cost and difficult production on large scales, due to tedious multistep reactions and complicated syntheses.^4,5^ Here, imperfect hyperbranched polymers can overcome the disadvantages of dendrimers because they are more conveniently produced (*e.g.*, through one-pot syntheses);^6^ accordingly, they are more popular than dendrimers in some applications (*e.g.*, sensor devices, special additive carriers, biology, blending, painting, coating).^3–7^

In general, two main approaches are used for the preparation of hyperbranched polymers: the single-monomer methodology, which involves polymerization of an AB_*n*_ monomer or an AB_*n*_ analogue, and the double-monomer methodology, which involves polymerization of two types of monomers or a monomer pair (*e.g.*, an A_2_ + B_*y*_ monomer combination), employing several common reactions (*e.g.*, ring-opening multi-branching polymerization; self-condensing vinyl polymerization; self-condensing ring-opening polymerization).^2,8^ Over the past two decades, much research effort has been dedicated to controlled syntheses for preparing hyperbranched polymers with precise degrees of branching (DBs),^9–16^ chemical conformations from linear to hyperbranched,^17–19^ DBs from 0 to 100%,^20^ and controlled molecular weights and distributions,^21–28^ in an effort to overcome the negative stigma associated with their perceived uncontrolled features. Hence, the development of well-defined and well-controlled hyperbranched polymers is an important step in their increased utility in academic studies and industrial applications.^3,29^

Benzoxazine (BZ) monomers possess the ability to undergo thermal ring-opening polymerization, without the need for any catalysts and without the formation of byproducts, forming polymers with several unique features (*e.g.*, high chemical resistance, low surface free energy, high flame retardancy) that have drawn the attention of both academia and industry.^30–37^ Triphenylamine (TPA) possesses a unique two-photon absorption ability and has been studied and applied in many fields, including optoelectronic devices and electron transition devices.^38–40^ Hence, we have been interested in combining the properties of BZ and TPA units in polymeric systems. In a previous study, we synthesized a series of BZ/TPA-based dendrimers [*e.g.*, a generation-three dendrimer (TPA–BZ DG3) possessing 22 TPA and 45 BZ units] through facile one-pot Mannich condensations.^41^ Interestingly, the small bathochromic shifts were observed in UV-Vis absorption and PL emission spectra upon increasing the number of TPA units, the result of an effective increase in the conjugation length.^42,43^ The symmetrical TPA–BZ dendrimers were constructed using TPA–BZ dendrons (asymmetrical hyperbranched monomers); we were interested in comparing the properties of the dendrons of symmetrical dendrimers and their corresponding hyperbranched monomers. The asymmetrical BZ monomers possess some properties that are very different from those of their symmetrical counterparts. Gu *et al.* reported that an asymmetrical monomer containing two BZ units exhibited characteristic signal splits in its ^1^H nuclear magnetic resonance (NMR; oxazine rings) and Fourier transform infrared (FTIR; vibrations of trisubstituted benzene ring at 930 cm^−1^) spectra.^44^ Endo *et al.* also found that linear asymmetrical monomers, solely containing various numbers of BZ units, exhibited diversity in their characteristic signal splits (*e.g.*, oxazine rings) in their ^1^H and ^13^C NMR spectra, depending on their chemical constructions.^45,46^ Hence, ^1^H and ^13^C NMR spectra can provide information about the chemical constructions of linear asymmetrical BZ monomers, due to the interactions of their adjacent groups, especially in the characteristic signal splits of the oxazine rings. We expected that the ^1^H and ^13^C NMR spectra of branching asymmetrical TPA–BZs would be affected more strongly than those of linear asymmetrical monomers because of the shorter distance between the adjacent groups; accordingly, it might be possible to monitor the complicated branching constructions of TPA–BZs in a very simple and straightforward approach. In addition, we have also investigated the photophysical properties of these asymmetrical hyperbranched TPA–BZ monomers.

**Scheme 1:**
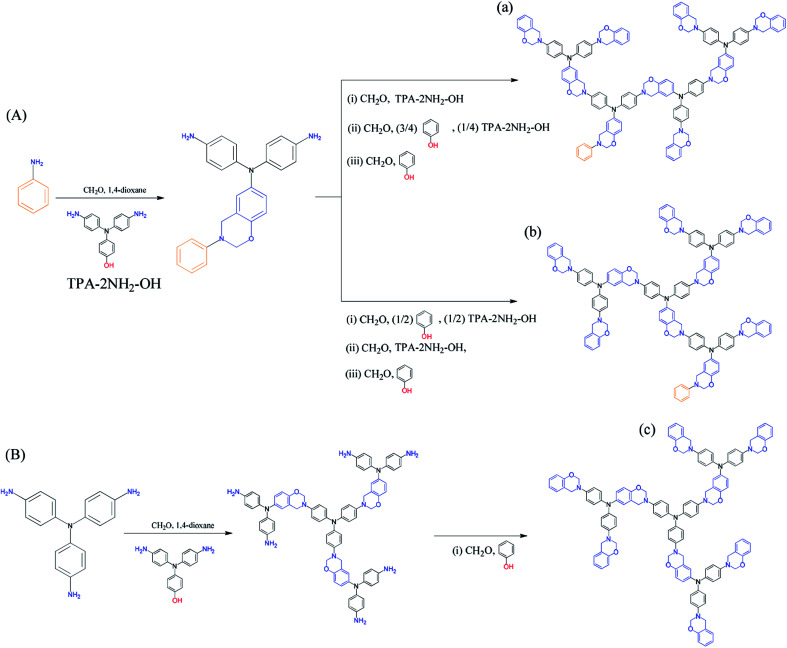
Synthesis of (A) the hyperbranched TPA–BZs (a) linear-tetramer and (b) tetramer and (B) the TPA–BZ dendrimer (c) TPA–BZ DG1.

**Scheme 2:**
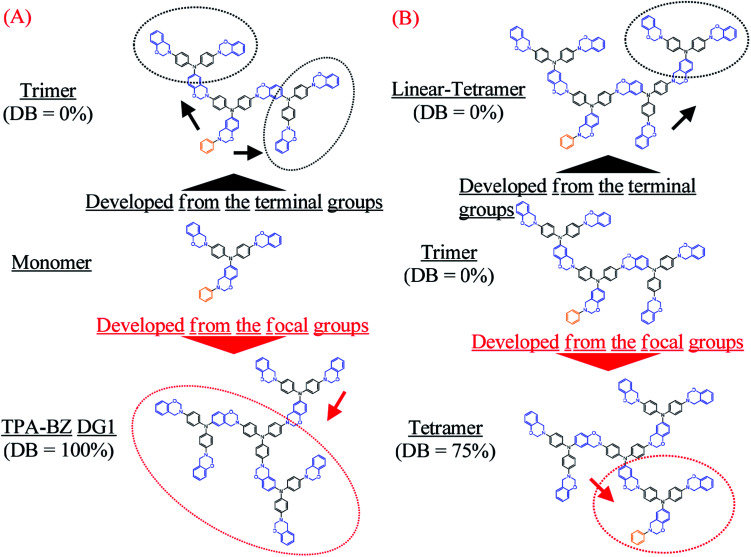
Directions of development from the terminal groups and the focal group in the chemical structures of the (A) monomer and (B) trimer.

In this study we synthesized a series of well-defined thermally polymerizable hyperbranched polymers (TPA–BZs), containing various numbers of TPA and BZ units, through facile one-pot Mannich condensations^41^ of TPA–2NH_2_–OH (as the AB_2_ branching groups), aniline (as the focal groups), CH_2_O, and phenol in 1,4-dioxane, using a unique feeding approach (Scheme 1 and S1† display this “click-like” reaction concept). To understand the different chemical construction and the conformation of these hyperbranched TPA–BZs, we used two design strategies including (i) simple hyperbranched TPA–BZs, such as those containing one or three TPA units (*i.e.*, TPA–BZ monomer and TPA–BZ trimer, respectively), developed from the focal or terminal group direction, as resultant monomers (Scheme 2); (ii) three dendritic TPA–BZs containing four TPA units (*i.e.*, linear TPA–BZ tetramer, TPA–BZ tetramer, TPA–BZ DG1) possessing different DBs (0–100%) for our conformation study.^47,48^ This work is the first study to incorporate BZ units into well-defined hyperbranched TPA-based polymers with various conformations (DBs from 0 to 100%) through such a convenient preparation strategy.

## Experimental section

### Materials

1-Fluoro-4-nitrobenzene (99%), hydrazine monohydrate (99%), trifluoroacetic anhydride (99%), *p*-anisidine (99%), cesium fluoride (99%), 10% Pd/C, charcoal, dimethyl sulfoxide (DMSO), dichloromethane, magnesium sulfate (MgSO_4_), and tetrahydrofuran (THF) were purchased from Alfa–Aesar. Aniline (99.5%), sodium borohydride (98%), boron tribromide (99.9%), sodium bicarbonate (NaHCO_3_), paraformaldehyde, ethyl acetate, methanol (MeOH), ethanol (EtOH), *n*-hexane, 1,4-dioxane, and phenol (99%) were purchased from Sigma–Aldrich. 4-(Bis(4-aminophenyl)amino)phenol (TPA–2NH_2_–OH) and TPA–BZ DG1 were prepared according to the literature.^41^

#### *N*,*N*-Bis(4-(2*H*-benzo[e][1,3]oxazin-3(4*H*)-yl)phenyl)-3-phenyl-3,4-dihydro-2*H*-benzo[*e*][1,3]oxazin-6-amine (TPA–BZ monomer)

A solution of CH_2_O (0.103 g, 3.43 mmol) and aniline (0.160 g, 1.72 mmol) in 1,4-dioxane (30 mL) was stirred under reflux at 90 °C for 2 h under N_2_ and then TPA–2NH_2_–OH (0.500 g, 1.72 mmol) was added. After 24 h under reflux, CH_2_O (0.206 g, 6.87 mmol) was added. After 2 h under reflux, phenol (0.323 g, 3.43 mmol) was added. After 24 h under reflux, the solution was cooled to room temperature and filtered. The filtrate was concentrated under reduced pressure and the residue taken up into CH_2_Cl_2_ (3 × 80 mL). The organic solution was washed several times with saturated aqueous NaHCO_3_ and distilled water, dried (MgSO_4_, 30 min), and concentrated. The residue was purified through column chromatography (SiO_2_; CH_2_Cl_2_/MeOH, 1 : 2) to yield a brown product (0.76 g, 69%): FTIR (KBr, cm^−1^): 1499, 1337, 1227, 941. ^1^H NMR (500 MHz, DMSO-*d*_6_, *δ*, ppm): 6.42–7.53 (m, aromatic CH), 5.36 and 5.42 (s, 2H, OCH_2_N), 4.58 and 4.63 (s, 2H, CCH_2_N). ^13^C NMR (125 MHz, DMSO-*d*_6_, *δ*, ppm): 153.96 (peak a), 151.14 (peak b), 147.82 (peak c), 143.63 (peak d), 115.24–153.96 (aromatic), 78.66 and 79.21 (OCH_2_N), 48.90 and 49.40 (CCH_2_N). MALDI-TOF MS: [M]^+^ (*m*/*z*) for (C_42_H_36_N_4_O_3_): 644.99: calc., 644.76 (Fig. S1†).

### TPA–BZ trimer

A solution of CH_2_O (0.0310 g, 1.03 mmol) and aniline (0.0480 g, 0.515 mmol) in 1,4-dioxane (30 mL) was stirred under reflux at 90 °C for 2 h under N_2_ and then TPA–2NH_2_–OH (0.150 g, 0.515 mmol) was added. After 24 h under reflux, CH_2_O (0.0620 g, 2.06 mmol) was added. After 2 h under reflux, TPA–2NH_2_–OH (0.300 g, 1.03 mmol) was added. After 24 h under reflux, CH_2_O (0.124 g, 4.12 mmol) was added. After 2 h under reflux, phenol (0.194 g, 2.06 mmol) was added. After 24 h under reflux, the solution was cooled to room temperature and filtered. The filtrate was concentrated under reduced pressure and the residue taken up into CH_2_Cl_2_ (3 × 80 mL). The organic solution was washed several times with saturated aqueous NaHCO_3_ and distilled water, dried (MgSO_4_, 30 min), and concentrated. The residue was purified through column chromatography (SiO_2_; CH_2_Cl_2_/MeOH, 1 : 2) to yield a brown product (0.55 g, 70%): FTIR (KBr, cm^−1^): 1499, 1337, 1227, 941. ^1^H NMR (500 MHz, DMSO-*d*_6_, *δ*, ppm): 6.39–7.55 (m, CH aromatic), 5.36 and 5.43 (s, 2H, OCH_2_N), 4.58 and 4.64 (s, 2H, CCH_2_N). ^13^C NMR (125 MHz, DMSO-*d*_6_, *δ*, ppm): 153.91 (peak a), 151.11 (peak b), 147.79 (peak c), 143.60 (peak d), 112.12–153.93 (aromatic), 78.63 and 79.18 (OCH_2_N), 48.87 and 49.36 (CCH_2_N).

### Linear TPA–BZ tetramer

A solution of CH_2_O (0.0260 g, 0.858 mmol) and aniline (0.0400 g, 0.429 mmol) in 1,4-dioxane (30 mL) was stirred under reflux at 90 °C for 2 h under N_2_ and then TPA–2NH_2_–OH (0.125 g, 0.429 mmol) was added. After 24 h under reflux, CH_2_O (0.0520 g, 1.72 mmol) was added. After 2 h under reflux, TPA–2NH_2_–OH (0.250 g, 0.858 mmol) was added. After 24 h under reflux, CH_2_O (0.103 g, 3.43 mmol) was added. After 2 h under reflux, phenol (0.121 g, 1.29 mmol) was added. After 24 h under reflux, TPA–2NH_2_–OH (0.125 g, 0.429 mmol) was added. After 24 h under reflux, CH_2_O (0.0520 g, 1.72 mmol) was added. After 2 h under reflux, phenol (0.0810 g, 0.858 mmol) was added. After 24 h under reflux, the solution was cooled to room temperature and filtered. The filtrate was concentrated under reduced pressure and the residue taken up into CH_2_Cl_2_ (3 × 80 mL). The organic solution was washed several times with saturated aqueous NaHCO_3_ and distilled water, dried (MgSO_4_, 30 min), and concentrated. The residue was purified through column chromatography (SiO_2_; CH_2_Cl_2_/MeOH, 1/2) to yield a brown product (0.54 g, 65%): FTIR (KBr, cm^−1^): 1499, 1339, 1227, 941. ^1^H NMR (500 MHz, DMSO-*d*_6_, *δ*, ppm): 6.43–7.29 (m, aromatic CH), 5.37 and 5.43 (s, 2H, OCH_2_N), 4.59 and 4.64 (s, 2H, CCH_2_N). ^13^C NMR (125 MHz, DMSO-*d*_6_, *δ*, ppm): 153.91 (peak a), 151.11 (peak b), 147.79 (peak c), 143.60 (peak d), 116.19–153.93 (aromatic), 78.63 and 79.18 (OCH_2_N), 48.87 and 49.36 (CCH_2_N).

### TPA–BZ tetramer

A solution of CH_2_O (0.0260 g, 0.858 mmol) and aniline (0.0400 g, 0.429 mmol) in 1,4-dioxane (30 mL) was stirred under reflux at 90 °C for 2 h under N_2_ and then TPA–2NH_2_–OH (0.125 g, 0.429 mmol) was added. After 24 h under reflux, CH_2_O (0.0260 g, 0.858 mmol) was added. After 2 h under reflux, phenol (0.0400 g, 0.429 mmol) was added. After 24 h under reflux, CH_2_O (0.0260 g, 0.858 mmol) was added. After 2 h under reflux, TPA–2NH_2_–OH (0.125 g, 0.429 mmol) was added. After 24 h under reflux, CH_2_O (0.0510 g, 1.72 mmol) was added. After 2 h under reflux, TPA–2NH_2_–OH (0.250 g, 0.858 mmol) was added. After 24 h under reflux, CH_2_O (0.103 g, 3.43 mmol) was added. After 2 h under reflux, phenol (0.162 g, 1.72 mmol) was added. After 24 h under reflux, the solution was cooled to room temperature and filtered. The filtrate was concentrated under reduced pressure and the residue taken up into CH_2_Cl_2_ (3 × 80 mL). The organic solution was washed several times with saturated aqueous NaHCO_3_ and distilled water, dried (MgSO_4_, 30 min), and concentrated. The residue was purified through column chromatography (SiO_2_; CH_2_Cl_2_/MeOH, 1 : 2) to yield a brown product (0.56 g, 67%): FTIR (KBr, cm^−1^): 1501, 1337, 1227, 941. ^1^H NMR (500 MHz, DMSO-*d*_6_, *δ*, ppm): 6.45–7.33 (m, aromatic CH), 5.37 and 5.44 (s, 2H, OCH_2_N), 4.59 and 4.65 (s, 2H, CCH_2_N). ^13^C NMR (125 MHz, DMSO-*d*_6_, *δ*, ppm): 153.91 (peak a), 151.11 (peak b), 147.79 (peak c), 143.61 (peak d), 115.21–153.91 (aromatic), 78.64 and 79.18 (OCH_2_N), 48.87 and 49.37 (CCH_2_N).

### TPA–BZ DG1

This compound was prepared using an approach described previously.^41^ A solution of TPA–3NH_2_ (0.130 g, 0.460 mmol) and CH_2_O (0.0820 g, 2.75 mmol) in 1,4-dioxane (30 mL) was stirred under reflux at 90 °C for 2 h under N_2_ and then TPA–2NH_2_–OH (0.400 g, 1.37 mmol) was added. After 24 h under reflux, CH_2_O (0.170 g, 5.49 mmol) was added. After 2 h under reflux, phenol (0.260 g, 2.75 mmol) was added. After 24 h under reflux, the solution was cooled to room temperature and filtered. The filtrate was concentrated under reduced pressure and the residue taken up into CH_2_Cl_2_ (3 × 80 mL). The organic solution was washed several times with saturated aqueous NaHCO_3_ and distilled water, dried (MgSO_4_, 30 min), and concentrated. The residue was purified through column chromatography (SiO_2_; CH_2_Cl_2_/MeOH, 1 : 2) to yield a brown product (0.56 g, 63%): FTIR (KBr, cm^−1^): 1499, 1339, 1225, 941. ^1^H NMR (500 MHz, DMSO-*d*_6_, *δ*, ppm): 6.50–7.22 (m, aromatic CH), 5.38 (s, 2H, OCH_2_N), 4.59 (s, 2H, CCH_2_N). ^13^C NMR (125 MHz, DMSO-*d*_6_, *δ*, ppm): 153.93 (peak a), 151.14 (peak b), 143.63 (peak d), 115.24–153.93 (aromatic), 79.22 (OCH_2_N), 49.40 (CCH_2_N).

### Thermal curing of hyperbranched TPA–BZ polymers

Desired amounts of the hyperbranched TPA–BZs were placed in Teflon dishes and thermally polymerized in a stepwise manner at 150, 180, 210, and 240 °C for 2 h each. Each cured sample had a brown-red color that became darker upon increasing the curing temperature.

### Characterization

^1^H and ^13^C NMR spectra were recorded using an INOVA 500 instrument with DMSO-*d*_6_ or CDCl_3_ as the solvent and tetramethylsilane as the external standard. FTIR spectra of the samples were recorded using a Bruker Tensor 27 FTIR spectrophotometer; 32 scans were collected at a spectral resolution of 4 cm^−1^. The prepared TPA–BZ sample films were sufficiently thin to obey the Beer–Lambert law. Mass spectra were recorded using a Bruker Daltonics Autoflex matrix-assisted laser desorption ionization-time of flight (MALDI-TOF) mass spectrometer. The following voltage parameter was used: ion source 1, 19.06 kV; ion source 2, 16.61 kV; lens, 8.78 kV; reflector 1, 21.08 kV; reflector 2, 9.73 kV. Dynamic curing kinetics were measured using a TA Q-20 differential scanning calorimeter operated under a N_2_ atmosphere. The TPA–BZ samples were placed into sealed aluminum sample pans. Dynamic curing scans were recorded from 25 to 320 °C at a heating rate of 20 °C min^−1^. The thermal stability of the TPA–BZ samples was determined using a TA Q-50 thermogravimetric analyzer. A thermally cured TPA–BZ sample was placed in a Pt cell and heated at a rate of 20 °C min^−1^ from 25 to 800 °C under a N_2_ atmosphere. UV-Vis spectra were recorded using a Shimadzu mini 1240 spectrophotometer; the concentration of the hyperbranched TPA–BZ (or TPA) in THF was 10^−4^ M. Photoluminescence (PL) excitation and emission spectra were recorded using a monochromatized Xe light source at room temperature.

## Results and discussion

### Preparation of four types of hyperbranched TPA–BZs and TPA–BZ DG1

Four well-defined hyperbranched TPA–BZs—TPA–BZ monomer, TPA–BZ trimer, linear TPA–BZ tetramer, and TPA–BZ tetramer (Scheme 1 and S1)—were synthesized from aniline (as the focal group), TPA–2NH_2_–OH (as the AB_2_ branching group), CH_2_O, and phenol in 1,4-dioxane through facile one-pot Mannich condensations with unique feeding sequences. The facile one-pot Mannich condensations employed formaldehyde/amine derivatives with uniquely activated terminal groups, rather than a strategy involving complicated and tedious protection/deprotection of functional groups.^41^ The mechanism of these Mannich condensations involved three steps (Scheme S2†); the first step was the reaction of a primary amine and CH_2_O to form a formaldehyde/amine derivative that could be used to activate the terminal groups of the primary amino groups of the TPA–BZ dendrimers or the hyperbranched TPA–BZs; the second and third steps were the direct reactions of the formaldehyde/amine derivatives with the phenol units from phenolic derivatives to form the BZ ring units; these phenolic derivatives include the AB_2_ branching groups of TPA–2NH_2_–OH and the terminal groups of phenol.^49,50^ We used the unique feeding sequences of these Mannich condensations to control the chemical constructions of the TPA–BZs with various DBs. Scheme S3† presents the possible mechanism of TPA–BZ trimer preparation, revealing how the feeding sequence of the reactants (aniline, TPA–2NH_2_–OH, CH_2_O, and phenol) was very important and strongly affected the chemical construction of the TPA–BZ; the concept is somewhat similar to building a structure from bricks of LEGO. The reactions of the formaldehyde/amine derivatives (*e.g.*, the reactions of the activated terminal groups of the hyperbranched TPA–BZs with phenolic derivatives such as TPA–2NH_2_–OH) in the first step of the Mannich condensation were “click-like” reactions (Scheme S2†). Three “click-like” reactions were used to prepare the TPA–BZ trimer (Scheme S3†); the first “click-like” reaction of the formaldehyde/amine derivative of aniline and the phenolic derivative of TPA–2NH_2_–OH is presented in Scheme S3(a);† the secondary and third are presented in Schemes S3(b) and S3(c),† respectively. The feeding sequence of the reactants could be used to control the chemical construction of the TPA–BZs through these “click-like” Mannich condensations. The feeding sequences for the preparation of the hyperbranched TPA–BZs are presented in Scheme 1 and S1.† TPA–BZ DG1 [Scheme 1(c)] was synthesized following an approach we have described previously.^41^

In our design strategy, the hyperbranched TPA–BZs were developed in the direction from the focal group or from the terminal groups of TPA–BZ monomer and TPA–BZ trimer (Scheme 2), respectively. Scheme 2A presents the two different directions for the chemical development of the TPA–BZ monomer—from its focal group to form the symmetrical TPA–BZ DG1 (downward) or from its terminal groups to form the asymmetrical TPA–BZ trimer (upward). Scheme 2B presents the two different directions for the chemical development of the TPA–BZ trimer—from its focal group to form the asymmetrical TPA–BZ tetramer (downward) or from its terminal groups to form the asymmetrical linear TPA–BZ tetramer (upward). ^1^H and ^13^C NMR, FTIR, and MALDI-TOF mass spectra^51–53^ were employed to investigate the chemical constructions and properties of the hyperbranched TPA–BZs.

### ^1^H NMR spectral analysis of hyperbranched TPA–BZs and TPA–BZ DG1

**Fig. 1:**
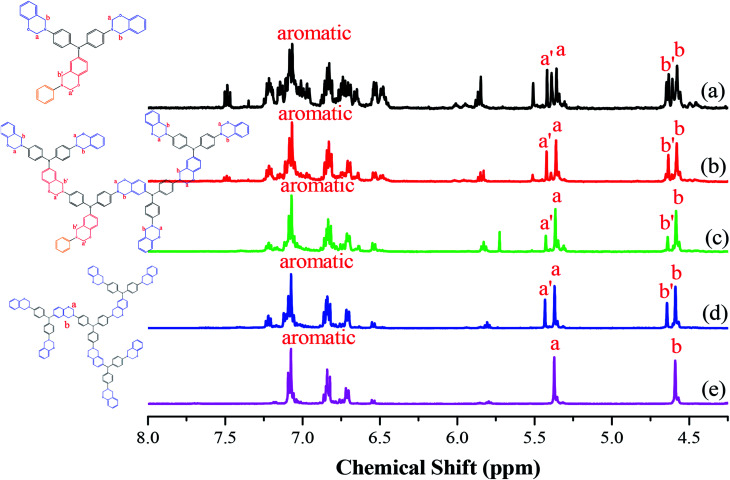
^1^H NMR spectra of the TPA–BZs: (a) monomer, (b) trimer, (c) linear-tetramer, (d) tetramer, and (e) TPA–BZ DG1.

Fig. 1 presents the ^1^H NMR spectra of the hyperbranched TPA–BZs and TPA–BZ DG1 in DMSO-*d*_6_ (the chemical constructions of the TPA–BZ trimer and TPA–BZ tetramer are displayed as Scheme S4†). Fig. 1a presents the significant splits of the characteristic signals of the O**CH**_**2**_N groups (5.28–5.53 ppm) for the TPA–BZ monomer; more than three peaks appeared that could be grouped into two portions, because of the asymmetrical chemical construction. The signals of the C**CH**_**2**_N groups (4.52–4.70 ppm) were similar to those of the O**CH**_**2**_N groups. Endo *et al.* reported that the number of characteristics signals (oxazine rings) in the ^1^H NMR spectra was consistent with the number of BZ groups for linear asymmetrical BZ monomers; these signal also tend to group into two portions, especially when more than three BZ units were present (namely BZ3 and BZ4).^45^ According to the number of signals (oxazine rings), the TPA–BZ monomer contained more than three BZ units (the theoretical number of the BZ units of TPA–BZ monomer), indicating that the signal splitting of the TPA–BZ monomer was more complicated than that of a linear asymmetrical monomer. The interactions of the protons of the oxazine rings and their adjacent groups were presumably influenced by the asymmetrical branching of the TPA–BZ monomer; that is, through interactions with the groups in both their main chains and branching chains.

Increasing the number of BZ units for the hyperbranched TPA–BZs [Fig. 1(b)–(d)] enhanced the tendency for signal splitting to two groups for both the O**CH**_**2**_N and C**CH**_**2**_N groups. For example, the peaks at 5.37 and 5.44 ppm (marked *a* and *a*′) corresponding to the O**CH**_**2**_N groups for TPA–BZ tetramer [Fig. 1d] featured two peaks, which we called OCH_2_N–L_1_ and OCH_2_N–H_1_, respectively. In addition, the peaks at 4.59 and 4.65 ppm (marked *b* and *b*′) correspond to C**CH**_**2**_N groups; herein, these two peaks are called CCH_2_N–L_2_ and CCH_2_N–H_2_, respectively. For the oxazine rings, the tendency for signal splitting into two portions for TPA–BZ tetramer was greater than that for TPA–BZ monomer.^45^ In contrast to the situation for the asymmetrical TPA–BZ tetramer, the ^1^H NMR spectrum of the symmetrical TPA–BZ DG1 (Fig. 1e) featured no signal splitting for the oxazine rings, with only two sharp signals appearing at 5.38 and 4.59 ppm (marked *a* and *b*) for the O**CH**_**2**_N (OCH_2_N–L_1_) and C**CH**_**2**_N (CCH_2_N–L_2_) units, respectively. These signal splits for the oxazine rings (marked *a*′ and *b*′) of the hyperbranched TPA–BZs were, therefore, related to their asymmetrical chemical constructions. Gu *et al.* proposed that an asymmetrical monomer containing two BZ units would feature a couple of characteristic signals in the ^1^H NMR spectrum for both the C**CH**_**2**_N and O**CH**_**2**_N groups. These signal are split as a result of the asymmetrical chemical construction.^44^ Endo *et al.* also reported that the ^1^H and ^13^C NMR spectra of a series of linear asymmetrical monomers would feature a diversity of characteristic signal splits for their oxazine rings depending on their various numbers of BZ units.^45^ As a result, the signal splits of oxazine rings can be affected by both the asymmetrical chemical constructions and the number of BZ units of the monomers.

**Fig. 2:**
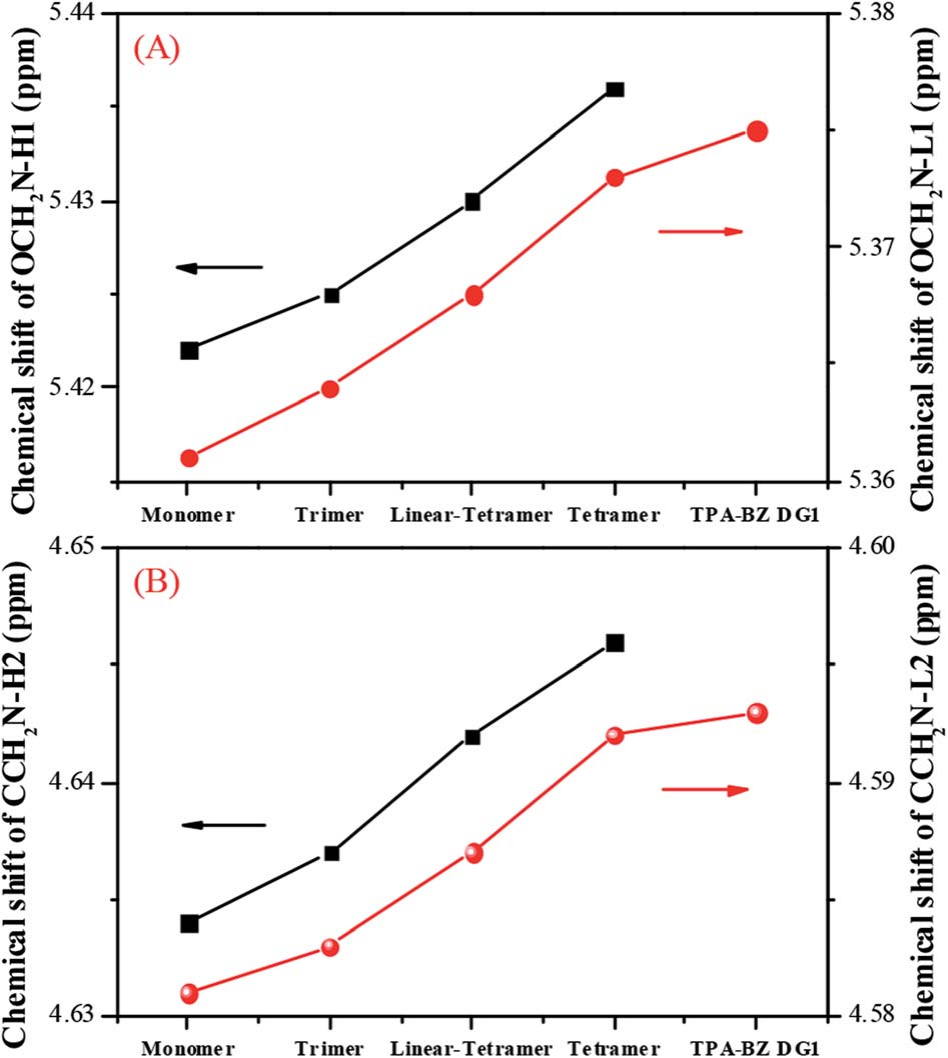
Chemical shifts in ^1^H NMR spectra of hyperbranched TPA–BZs and TPA–BZ DG1: (A) OCH_2_N–H1 and OCH_2_N–L1; (B) CCH_2_N–H2 and CCH_2_N–L2.

Fig. 2A presents the chemical shifts of the signals for OCH_2_N–H_1_ and OCH_2_N–L_1_ in the ^1^H NMR spectra of the hyperbranched TPA–BZs and TPA–BZ DG1. Both chemical shifts increased upon proceeding from small to large numbers of TPA units and DBs: TPA–BZ monomer, TPA–BZ trimer, linear TPA–BZ tetramer, TPA–BZ tetramer, and TPA–BZ DG1. Fig. 2B presents the chemical shifts of the signals for CCH_2_N–H_2_ and CCH_2_N–L_2_ in the ^1^H NMR spectra of the hyperbranched TPA–BZs and TPA–BZ DG1; the results are similar to those in Fig. 2A. Hence, we conclude that these chemical shifts (OCH_2_N–H_1_, OCH_2_N–L_1_, CCH_2_N–H_2_, and CCH_2_N–L_2_) are enhanced upon increasing the number of TPA units and DBs of the TPA–BZ monomers. For the signals of the oxazine rings in the ^1^H NMR spectra, Table 1 summarizes the chemical shifts and the proportions of integrated areas (signal splits) for the hyperbranched TPA–BZs and TPA–BZ DG1.

**Table 1:** Calculation results of *H*_BZ_ and *L*_BZ_ in ^1^H NMR spectra for hyperbranched TPA–BZs and TPA–BZ DG1^a^

Sample		Monomer	Trimer	Linear-tetramer	Tetramer	TPA–BZ DG1
OCH_2_N–H1	*δ*, ppm	5.422	5.425	5.43	5.436	—
	Area (%)	37.2	41.3	22.0	34.5	0
OCH_2_N–L1	*δ*, ppm	5.361	5.364	5.368	5.373	5.375
	Area (%)	62.8	58.7	78.0	65.5	100.0
CCH_2_N–H2	*δ*, ppm	4.634	4.637	4.642	4.646	—
	Area (%)	33.1	42.0	22.7	31.8	0
CCH_2_N–L2	*δ*, ppm	4.581	4.583	4.587	4.592	4.593
	Area (%)	66.9	58.0	77.3	68.2	100.0
BZ grouping	BZ number	3*	7*	9*	9*	9*
	*H*_BZ_	1.05 (1*)	2.92 (3*)	2.01 (2*)	2.98 (3*)	0.00 (0*)
	*L*_BZ_	1.95 (2*)	4.08 (4*)	6.99 (7*)	6.02 (6*)	100.00 (9*)

a “*” indicates the theoretical numbers of the BZ units.

The asymmetrical linear BZ monomer feature a number of intramolecular cross-interactions among its oxazine moieties and aromatic hydrogen atoms, thereby affecting the proton resonances of the oxazine rings, resulting in their signal splitting in ^1^H NMR spectra; for example, the signals of the oxazine moiety connected directly to the focal group (benzyl group) tended to be located downfield in the ^1^H NMR spectrum, comparing with those of the other oxazine rings in the monomer.^45^ The chemical shifts of oxazine rings depend on their relative locations in the asymmetrical BZ monomers; they tended to merge into two significant portions, representing the two groups of different proton resonances of the oxazine rings, as the sample concentration increased, with the resulting aggregation enhancing the intramolecular cross-interactions^45^ by shortening the distances the between oxazine moieties and the aromatic hydrogen atoms. The BZ units in the TPA–BZ monomers served connection or terminal functions; they all existed in the branching chemical constructions (the AB_2_ branching groups) and were very close to their adjacent groups, especially in those containing higher numbers of TPA and BZ units, potentially enhancing the intramolecular cross-interactions and the separation into two significant portions. The integrated areas of these two portions could be used to analyze the relationships among the characteristic signal splits and the chemical constructions of the TPA–BZ monomers.

The ratios of the integrated areas of the OCH_2_N/CCH_2_N signals (oxazine rings) in the ^1^H NMR spectra of TPA–BZ monomer, TPA–BZ trimer, linear TPA–BZ tetramer, TPA–BZ tetramer, and TPA–BZ DG1 were 2.39/2.63, 1.84/2.00, 2.00/2.11, 2.00/2.17, and 2.00/2.09, respectively; these values are all close to 1.00, suggesting high purity for the hyperbranched TPA–BZs. Table 1 presents the integrated area proportions of the two portions of signals for the OCH_2_N and CCH_2_N groups; for example, the integrated area proportions of OCH_2_N–H_1_ and OCH_2_N–L_1_ for TPA–BZ tetramer were 34.5 and 65.5%, respectively, while those of CCH_2_N–H_2_ and CCH_2_N–L_2_ were 31.8 and 68.2%, respectively. The integrated area proportions for OCH_2_N–H_1_ and CCH_2_N–H_1_ were very close. Therefore, the number of BZ units providing downfield (*H*_BZ_) signals in the ^1^H NMR spectra could be calculated using the equation (theoretical number of BZ units of the hyperbranched TPA–BZs) × (average integrated area proportion of OCH_2_N–H_1_ and CCH_2_N–H_1_ for the hyperbranched TPA–BZs)/100. The number of BZ groups providing upfield (*L*_BZ_) signals in the ^1^H NMR spectra was calculated using the equation (theoretical number of BZ units of the hyperbranched TPA–BZs) – (*H*_BZ_ of the hyperbranched TPA–BZs). For example, the value of *H*_BZ_ of TPA–BZ tetramer was 2.98 = [9 × (34.5 + 31.8)/2/100]; the value of *L*_BZ_ of TPA–BZ tetramer was 6.02 = [9 − 2.98]. These values for TPA–BZ tetramer are very close to three and six, respectively, suggesting that there were three BZ units (*H*_BZ_ = 3) that were possibly affected by the asymmetrical chemical construction and that their signal splits were located downfield in the ^1^H NMR spectra, comparing with those of *L*_BZ_. These three BZ units were presumably positioned in some unique locations in the chemical constructions of TPA–BZ tetramer. Such *H*_BZ_ analysis can possibly be used to investigate the chemical constructions of hyperbranched TPA–BZs. Table 1 presents the calculated number of BZs, and the values of *H*_BZ_ and *L*_BZ_, in the ^1^H NMR spectra of the hyperbranched TPA–BZs.

The characteristic signal splits (oxazine rings) in the ^1^H NMR spectra for the linear asymmetrical monomers resulted from the protons of the oxazine rings interacting with adjacent aromatic hydrogen atoms, based on an analysis using ^1^H–^1^H NOESY 2D NMR spectroscopy, because the chemical constructions of the monomer featured a BZ ring group with a diversity of connected components at its two ends: including BZ rings or a benzyl group (the focal group) or empty (a hydrogen atom).^45^ The chemical constructions of the hyperbranched TPA–BZs or TPA–BZ DG1 featured a >*N*-(benzyl-BZ)-*N*< unit, which had a benzyl BZ group (b-BZ set) with a diversity of connected components at its two ends (*e.g.*, the nitrogen atoms with their connected b-BZ sets), depending on the role served by the b-BZ set in the TPA–BZ monomer (*e.g.*, focal, connection, or terminal function). Therefore, the characteristic signal splits (oxazine rings) of the b-BZ set in the ^1^H NMR spectra of hyperbranched TPA–BZs and TPA–BZ DG1 were possibly affected by the adjacent b-BZ sets connected to the nitrogen atoms at its two ends (intramolecular cross-interactions); thus, it might possibly be used to analyze the relationships between the signal splitting and the chemical constructions of the hyperbranched TPA–BZs.

Consequently, we selected a piece of the structural composition, which possessed a b-BZ set with its two ends connected to components (*e.g.*, the nitrogen atoms and their connected b-BZ sets), to analyze the proton resonances of the oxazine rings in the ^1^H NMR spectra (*H*_BZ_ and *L*_BZ_ portions) for the TPA–BZs. The structural compositions of the hyperbranched TPA–BZs and the TPA–BZ DG1 were analyzed and sorted in terms of the roles served by the b-BZ and its two end-connected compositions (composed by several b-BZ sets and nitrogen atoms) components (Table S1†). The code rules of the structural in this study are presented in Tables S1 and S4:† (i) the arrow in red indicates the position of the designated BZ ring (^1^H NMR spectra) or the designated carbon atom (^13^C NMR spectra); (ii) the numbers 1, 2, 2̲, and 3 represent the b-BZ sets serving the focal function, the connection function, the connection function featuring a connected component of a core group of the TPA–BZ dendrimer, and the terminal function, respectively; (iii) the focal composition of [*Fxx*]: “*F*” indicates the focal composition and “*xx*” indicates that the b-BZ set of the focal function has two b-BZ sets at its end nitrogen atom close to the BZ ring (*e.g.*, [F33] indicates that one focal function set has two terminal function sets at its end nitrogen atom closed to the BZ ring); (iv) the connection composition of [*Cxxyy*]: “*C*” indicates the connection composition, “*xx*” indicates that the b-BZ set of the connection function has two b-BZ sets at its end nitrogen atom close to the BZ ring, and “*yy*” indicates that the b-BZ set of the connection function has two b-BZ sets at its end nitrogen atom close to the benzyl group (*e.g.*, [C3312] indicates that one connection function set has two terminal function sets at its end nitrogen atom close to the BZ ring, and one focal and one connection function set at its end nitrogen atom close to the benzyl group); (v) the terminal composition of [*Txx*]: “*T*” indicates the terminal composition and “*xx*” indicates that the b-BZ set of the terminal function has two b-BZ sets at its end nitrogen atom close to the benzyl group (*e.g.*, [T12] indicates that one terminal function set has one focal and one connection function set at its end nitrogen atom close to the benzyl group).

We used 14 types of structural compositions (Table S1†) to analyze the signals of *H*_BZ_ and *L*_BZ_ in the ^1^H NMR spectra of the hyperbranched TPA–BZs and TPA–BZ DG1; for example, TPA–BZ trimer was composed of three types of structural compositions: one piece of [F22], two pieces of [C3312], and four pieces of [T23]. These structural compositions could be used to analyze the relationship between the chemical construction and the portions of *L*_BZ_ and *H*_BZ_, which represented the characteristic signal splits of the oxazine rings in the downfield region of the ^1^H NMR spectra. We employed a systematic methodology using the 14 types of structural compositions to analyze the relationship between the *H*_BZ_ portion and the chemical constructions of the hyperbranched TPA–BZs and TPA–BZ DG1. Table S1† reveals the six types of structural compositions possibly belonging to the characteristic signal splits of the oxazine rings in the downfield region of the ^1^H NMR spectra (marked *H*_BZ_): [F33], [F23], [F22], [C3312], [C2213], and [T12]. In other words, the chemical constructions of TPA–BZ monomers possessing these six types of structural compositions would possibly lead to the characteristic signal splits (oxazine rings) observed in the downfield region of the ^1^H NMR spectra; for example, there are three types of structural compositions of TPA–BZ tetramer belonging to the *H*_BZ_ portion (*H*_BZ_ = 3): one piece of [F23], one piece of [C2213], and one piece of [T12]. The analytical results for the values of *H*_BZ_ and *L*_BZ_ in Table S1† are consistent with the calculated results determined from the ^1^H NMR spectra in Table 1. These six types of structural compositions could be used to analyze the relationship between the *H*_BZ_ portion in the ^1^H NMR spectra and the chemical constructions of the hyperbranched TPA–BZs and TPA–BZ dendrimers.

**Scheme 3:**
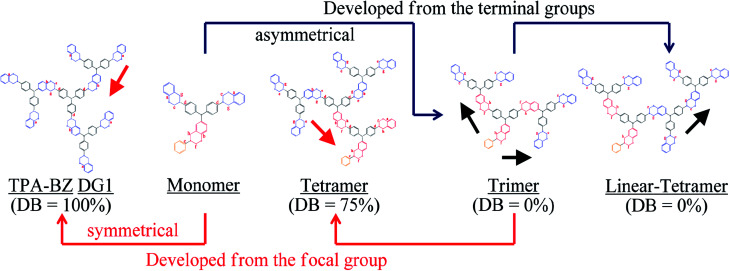
Possible mechanism of *H*_BZ_, *L*_BZ_, and *C*_f_ analysis for hyperbranched TPA–BZs and TPA–BZ DG1.

Scheme 3 presents the possible mechanism of *H*_BZ_ and *L*_BZ_ portion analysis for the hyperbranched TPA–BZs and TPA–BZ DG1; the BZ rings in red and blue belong to the *H*_BZ_ and *L*_BZ_ portions, respectively, in the ^1^H NMR spectra. The values of *H*_BZ_ and *L*_BZ_ are summarized in Table 2. For hyperbranched TPA–BZs, the b-BZ set of the focal function (*e.g.*, [F33], [F23], and [F22]) and the b-BZ sets connected at its end nitrogen atom close to the BZ ring (*e.g.*, the connection function sets of [C3312] and [C2213], and terminal function set of [T12]) belong to the *H*_BZ_ portion, except in the connection function set of [C2312] for linear TPA–BZ tetramer. As a result, the characteristic signal splits (oxazine rings) in the ^1^H NMR spectra are affected strongly by the focal group (benzyl group). All BZ rings (all structural compositions) of TPA–BZ DG1 belong to the *L*_BZ_ portion, due to its symmetrical chemical construction. For the TPA–BZs containing four TPA groups, the *H*_BZ_ can be used to distinguish the conformations of the TPA–BZs (DBs from 0 to 100%), even when these compounds possess the same molecular weight; for example, the values of *H*_BZ_ of linear-tetramer (DB = 0%), tetramer (DB = 75%), and TPA–BZ DG1 (DB = 100%) were two, three, and zero, respectively (Table 2). Therefore, these six structural compositions of the *H*_BZ_ portion in the ^1^H NMR spectra could be used as a powerful tool to investigate the chemical constructions of hyperbranched and dendrimer TPA–BZs, especially for those with higher numbers of TPA and BZ units and various DBs.

**Table 2:** Sample information and values of *H*_BZ_, *L*_BZ_, and *C*_f_ of the hyperbranched TPA–BZs and TPA–BZ DG1^a^

Compound		TPA–BZ DG1	Monomer	Tetramer	Trimer	Linear-tetramer
DB (%)		100	—	75	0	0
Type		Dendrimer	Hyperbranched	Hyperbranched	Hyperbranched	Hyperbranched
TPA group		4	1	4	3	4
BZ group		9	3	9	7	9
^1^H NMR	*H*_BZ_	0	1	3	3	2
	*L*_BZ_	9	2	6	4	7
^13^C NMR *C*_f_ (ppm)	*P*_a_	0.029	0.058	0.007	0	0
	*P*_b_	0.037	0.029	0.007	0	0
	*P*_c_	—	0.029	0.007	0	0
	*P*_d_	0.029	0.029	0.007	0	0
	*P*_e_	0.036	0.029	0	0	0
	*P*_f_	—	0.029	0.007	0	0
	*P*_g_	0.037	0.037	0.007	0	0
	*P*_h_	—	0.029	0	0	0

a The *H*_BZ_, *L*_BZ_, and *C*_f_ (*P*_e_–*P*_h_) belong to the characteristic signals of oxazine rings.

The design strategies of the TPA–BZ chemical constructions (Scheme 2) were also used to analyze the relationship between the value of *H*_BZ_ and the chemical construction developments of TPA–BZ monomer and TPA–BZ trimer. Scheme 3 and Table S2† present the relationships between the value of *H*_BZ_ and their chemical construction developments as the number of TPA or BZ units increased for TPA–BZ monomer and TPA–BZ trimer. TPA–BZ DG1 comprised three TPA–BZ monomers developed from their focal groups connected to the same center nitrogen atom, and the value of *H*_BZ_ of TPA–BZ monomer decreased from one to zero as a result of the symmetrical dendrimer formation of TPA–BZ DG1. The linear asymmetrical TPA–BZ trimer was developed from the terminal groups of TPA–BZ monomer by adding two single AB_2_ branching groups of TPA with the BZ terminal groups connected to TPA–BZ monomer's two terminal groups; the value of *H*_BZ_ of TPA–BZ monomer increased from one to three. The branching asymmetrical TPA–BZ tetramer was developed from the focal group of TPA–BZ trimer, which connected to one of the terminal groups of the single AB_2_ branching group of TPA containing one focal group and one BZ terminal group; the value of *H*_BZ_ of TPA–BZ trimer decreased from three to one. The value of *H*_BZ_ of TPA–BZ tetramer, however, was also three; it belonged to the three b-BZ sets connected to the center nitrogen atom containing a b-BZ set of focal function. The linear asymmetrical linear TPA–BZ tetramer was developed from the terminal group of the trimer by adding a single AB_2_ branching group of TPA containing two BZ terminal groups connected to one of the terminal groups of TPA–BZ trimer; the value of *H*_BZ_ of TPA–BZ trimer decreased from three to two.

The BZ groups (the b-BZ sets) of the *H*_BZ_ portion were usually located in the connected components at the end nitrogen atom of the focal function set. Interestingly, the value of *H*_BZ_ of TPA–BZ trimer decreased after the addition of a TPA group connected to its one terminal group, resulting in the formation of the linear asymmetrical linear TPA–BZ tetramer. Hence, the linear asymmetrical TPA–BZ monomer (DB = 0%) developed from the terminal groups of TPA–BZ trimer possibly led to the decrease in the value of *H*_BZ_. The values of *H*_BZ_ for the TPA–BZ monomers in this study ranged from zero to three; a value of zero indicates that the TPA–BZ monomer belongs to the symmetrical dendrimer; a value of *H*_BZ_/*L*_BZ_ of 1/2 indicates that the TPA–BZ is the monomer; a value of *H*_BZ_/*L*_BZ_ of 2/7 indicates that the TPA–BZ is the linear-tetramer; a value of *H*_BZ_/*L*_BZ_ of 3/4 indicates that the TPA–BZ is the trimer; a value of *H*_BZ_/*L*_BZ_ of 3/6 indicates that the TPA–BZ is the tetramer. Hence, the pairs of values of *H*_BZ_ and *L*_BZ_ can be used conveniently to identify the chemical constructions and conformations of the TPA–BZs.

### ^13^C NMR spectral analysis of hyperbranched TPA–BZs and TPA–BZ DG1

**Fig. 3:**
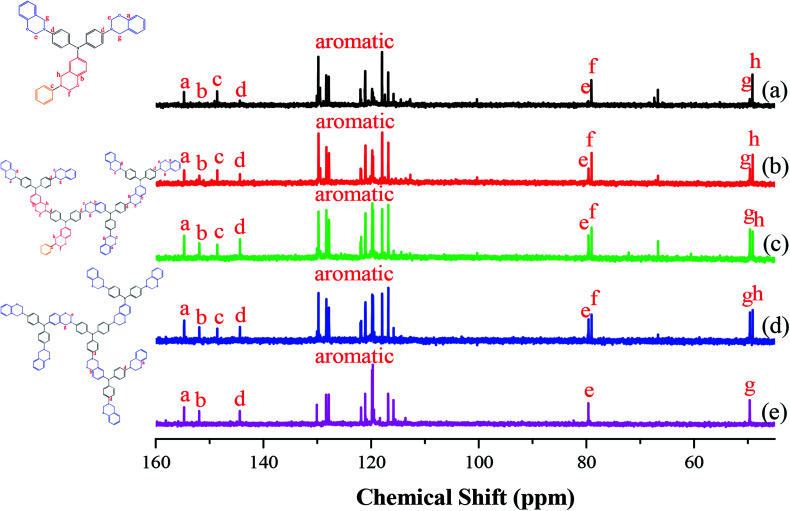
^13^C NMR spectra of the TPA–BZs: (a) monomer, (b) trimer, (c) linear-tetramer, (d) tetramer, and (e) TPA–BZ DG1.

Fig. 3 presents the ^13^C NMR spectra of the hyperbranched TPA–BZs and TPA–BZ DG1 in DMSO-*d*_6_ (the chemical constructions of the annotated ^13^C NMR spectra for TPA–BZ trimer and TPA–BZ tetramer are displayed in Scheme S5†). Fig. S2† provides an enlarged view of the ^13^C NMR spectrum of TPA–BZ monomer (Fig. 3a) from 138 to 168 ppm. Fig. 3(a)–(c) present the ^13^C NMR spectra of TPA–BZ monomer, TPA–BZ trimer, and linear TPA–BZ tetramer, respectively, including four characteristic signals in the range from 143 to 154 ppm (peaks a–d) and four characteristic signals of oxazine rings in the range from 48 to 80 ppm (peaks e–h), including two characteristic signal splits (peak f and h) arising from the asymmetrical chemical constructions, similar to the situation in the ^1^H NMR spectra. Fig. 3d presents the ^13^C NMR spectrum of TPA–BZ tetramer; peak a (namely *P*_a_; 153.912 ppm) corresponds to the aromatic CH_2_O**C** nuclei of the b-BZ sets of the terminal function; peak b (namely *P*_b_; 151.113 ppm) corresponds to the aromatic CH_2_O**C** nuclei of the b-BZ sets of the connection and focal functions; peak c (namely *P*_c_; 147.794 ppm) corresponds to the aromatic CH_2_N**C** nuclei of the b-BZ sets of the focal functions; peak d (namely *P*_d_; 143.611 ppm) corresponds to the aromatic CH_2_N**C** nuclei of the b-BZ sets of connection and terminal functions; peaks e and f (namely *P*_e_ and *P*_f_; 79.183 and 78.640 ppm, respectively) correspond to O**C**H_2_N nuclei of the oxazine rings; and peaks g and h (namely *P*_g_ and *P*_h_; 49.371 and 48.866 ppm) correspond to the C**C**H_2_N nuclei of the oxazine rings. The chemical shifts of the characteristic signals in the ^13^C NMR spectra of the hyperbranched TPA–BZs and TPA–BZ DG1 are summarized in Table S3.†Fig. 3e presents the ^13^C NMR spectrum of TPA–BZ DG1 in DMSO-*d*_6_. *P*_a_ and *P*_b_ (153.934 and 151.143 ppm, respectively) correspond to the aromatic CH_2_O**C** nuclei of the b-BZ sets of the terminal and the connection functions, respectively. *P*_d_ (143.633 ppm) corresponds to the aromatic CH_2_N**C** nuclei of the b-BZ sets of the connection and terminal functions. *P*_e_ (79.219 ppm) corresponds to O**C**H_2_N nuclei of the oxazine rings. *P*_g_ (49.401 ppm) corresponds to C**C**H_2_N nuclei of the oxazine rings. TPA–BZ DG1 has no signal *P*_c_ because the dendrimer lacked a focal group (benzyl group). The spectrum of TPA–BZ DG1 features only one signal for each O**C**H_2_N and C**C**H_2_N nucleus (*P*_e_ and *P*_g_, respectively). In contrast, the spectrum of TPA–BZ tetramer has two signals for each O**C**H_2_N (*P*_e_ and *P*_f_) and C**C**H_2_N (*P*_g_ and *P*_h_) nucleus, due to its asymmetrical chemical construction. Hence, the asymmetrical chemical construction of a TPA–BZ enhances the tendency of the characteristic signal splits (*P*_f_ and *P*_h_) of the oxazine rings in the upfield region of the ^13^C NMR spectra, compared with that of their symmetrical counterparts; the behavior is similar to that of linear BZ monomers.^45^ Interestingly, the characteristic signal splits of the oxazine rings for TPA–BZs arising from the asymmetrical chemical construction appear downfield and upfield, compared with those of the symmetrical counterparts, in the ^1^H and ^13^C NMR spectra, respectively.

**Fig. 4:**
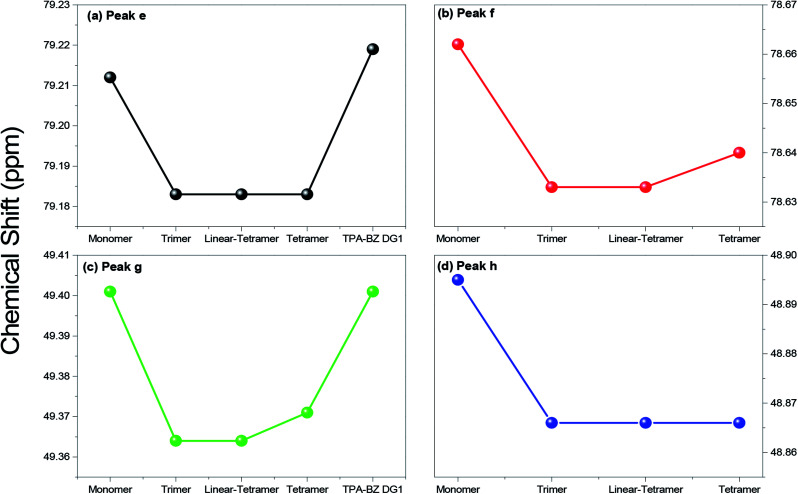
Chemical shifts in ^13^C NMR spectra of hyperbranched TPA–BZs and TPA–BZ DG1 for peaks (a) e, (b) f, (c) g, and (d) h.

Fig. S3† presents the chemical shifts of *P*_a_–*P*_d_ in the ^13^C NMR spectra of the hyperbranched TPA–BZs and TPA–BZ DG1; the distribution of these data for each peak followed a similar tendency of three steps of (i) decreasing, (ii) keeping constant, and (iii) increasing, as the sample order changed from TPA–BZ monomer, trimer, linear-tetramer, tetramer, to TPA–BZ DG1. Fig. 4 presents the chemical shifts P_e_–P_h_ of the oxazine rings in the ^13^C NMR spectra of the hyperbranched TPA–BZs and TPA–BZ DG1, revealing similar data distributions of the three steps as those in Fig. S3† (*P*_a_–*P*_d_); the situation is very different from that in the ^1^H NMR spectra. Interestingly, the chemical shift of each peak *P*_a_–*P*_h_ for TPA–BZ trimer and linear TPA–BZ tetramer remained unchanged at a relative lower constant, comparing with those of the other three monomers in this study, indicating that these eight chemical shifts for these linear TPA–BZ monomers (DB = 0%) were possibly not affected by the effect of the number of TPA or BZ groups.

The ^13^C NMR spectral data for the linear asymmetrical monomers BZ2, BZ3, and BZ4^45^ revealed four characteristic signal portions in the range from 142.5 to 154.4 ppm, representing the aromatic carbon nuclei of the CH_2_O**C** and CH_2_N**C** groups, and four characteristic signal portions in the range from 50.5 to 80.5 ppm, representing the O**C**H_2_N and C**C**H_2_N groups; the mergence extension for these eight signal portions possibly can be enhanced by using the solution concentration enhancement, resulting in the intramolecular cross-interactions increment. As a result, these eight signal portions also tended to keep at an approximate constant chemical shift for each signal portion, similar to the behavior of *P*_a_–*P*_h_ for the linear TPA–BZ trimer and linear-tetramer. These eight signals (*P*_a_–*P*_h_) of the hyperbranched TPA–BZs exhibited well mergence extension for each signal, indicative of enhanced intramolecular cross-interactions, possibly resulting from shorter distances between the designated carbon atoms and their adjacent groups, due to their branched chemical structures (AB_2_ branching groups), compared with those of the linear asymmetrical BZ monomers, similar to the behavior in the ^1^H NMR spectra. Interestingly, these characteristic signals (portions) of the linear asymmetrical monomers for hyperbranched TPA–BZs and all tended to remain approximately constant chemical shift, and were not affected by the incorporation of the TPA groups into the monomers or the branched chemical structures; this phenomenon can, therefore, be used as a standard or base when using ^13^C NMR spectroscopy to analyze the chemical structures or conformations of asymmetrical TPA–BZ monomers. In addition, these eight characteristic signals in the ^13^C NMR spectra are affected strongly upon changing the relative position of the attached oxazine ring in the monomer [*e.g.*, in the *para*, *meta*, or *ortho* position of the linear asymmetrical monomer solely containing two BZ moieties (BZ2)].^46^ These chemical shifts (*P*_a_–*P*_h_) for TPA–BZ monomer and TPA–BZ DG1 appear at relatively higher values compared with those of TPA–BZ tetramer. The chemical shifts (*P*_a_–*P*_h_) of TPA–BZ tetramer are located between those of TPA–BZ DG1 and linear TPA–BZ tetramer; these three monomers have the same molecular weight, but different chemical conformations (Table 2). As a result, the eight chemical shifts (*P*_a_–*P*_h_) of the monomers containing four TPA groups and night BZ groups should shift downfield as the DB increases.

According to this analysis of the eight signals *P*_a_–*P*_h_, the linear TPA–BZs (trimer and linear-tetramer featuring DB = 0%) exhibited the most upfield chemical shifts that remained unchanged for each peak, relative to those of the other three monomers in this study. We suspect that the chemical conformations of the TPA–BZ monomers affected these chemical shifts (*P*_a_–*P*_h_). The eight chemical shifts (*P*_a_–*P*_h_) of linear-tetramer could be used as a base for examining those of the other monomers in this study. We define the value of *C*_f_ (ppm) in the ^13^C NMR spectrum for peak *x* (namely P_*x*_; *x* = a–h) as [(chemical shift of P_*x*_ of TPA–BZ monomer) – (chemical shift of P_*x*_ of linear-tetramer)]. Table 2 summarizes the values of *C*_f_ in the ^13^C NMR spectra of the hyperbranched TPA–BZs and TPA–BZ DG1. The chemical shifts (*P*_a_–*P*_h_) were affected by the chemical constructions and conformations, especially by the DB. Hence, the values of *C*_f_ were more suitable than the chemical shifts alone when evaluating the chemical constructions and conformations of the TPA–BZ monomers; that is, the differences in their chemical constructions could be investigated more clearly.

Fig. S4† presents the values of *C*_f_ of *P*_a_–*P*_d_ for the hyperbranched TPA–BZs and TPA–BZ DG1. For TPA–BZ monomer, these values were higher than those of the other hyperbranched TPA–BZs, especially for *P*_a_ (0.058 ppm). The values of *C*_f_ (*P*_a_–*P*_d_) of TPA–BZ trimer all remained at a constant at zero, due to the linear structure of TPA–BZ monomer (DB = 0%). The values of *C*_f_ for signals *P*_a_, *P*_b_, and *P*_d_ of TPA–BZ DG1 were higher than those of the other hyperbranched TPA–BZs, especially in terms of *P*_b_ (0.037 ppm). The values of *C*_f_ of *P*_a_–*P*_d_ of TPA–BZ tetramer were all the same (0.007 ppm). Interestingly, the lowest values of *C*_f_ of *P*_a_–*P*_d_ for TPA–BZ monomer and TPA–BZ DG1 all remained constant at 0.029 ppm.

According to the concept of *H*_BZ_ and *L*_BZ_ portion analysis, the structural compositions could be selected to analyze the relationship between the *H*_BZ_ portion and the chemical constructions (conformations), using ^1^H NMR spectra. For the ^13^C NMR spectra, we selected a structural composition possessing a b-BZ set and its two end-connected components (*e.g.*, nitrogen atoms and their connected b-BZ sets) to analyze the characteristic signals of *P*_a_–*P*_c_, and selected a structural composition of *t*-BZs containing three b-BZ sets (their one end connected to the same center nitrogen atom) to analyze the characteristic signal of *P*_d_. Table S4† summarizes the structural compositions of the hyperbranched TPA–BZs and TPA–BZ DG1 analyzed in terms of peaks *P*_a_–*P*_d_.

For hyperbranched TPA–BZs and TPA–BZ DG1, 14 among the 25 types of structural compositions possibly had values of *C*_f_ (*P*_a_–*P*_d_) greater than zero (*C*_fs_ > 0) in their ^13^C NMR spectra, based on the analytical results in Table S4 (code rules and analysis progresses are provided in the ESI†). Scheme 3 presents the possible mechanism behind this phenomenon for hyperbranched TPA–BZs and TPA–BZ DG1. The carbon atoms in the chemical structures of the TPA–BZ monomers labeled with a letter *x* (*x* = a, b, c, or d) are those that possibly affected the positive value of *C*_f_ for peak *x* (P_*x*_). The *C*_fs_ > 0 (*P*_a_–*P*_d_) of TPA–BZ monomer were possibly affected by the structural compositions of [T13] (*P*_a_, 0.058 ppm), [F33] (*P*_b_, 0.029 ppm), [F33] (*P*_c_, 0.029 ppm), and [U133] (*P*_d_, 0.029 ppm). TPA–BZ trimer has no structural components that affected its *C*_f_ > 0, due to its linear TPA–BZ monomers. The *C*_fs_ > 0 of TPA–BZ tetramer were possibly affected by the structural compositions of [T12] (*P*_a_, 0.007 ppm), [C2213] (*P*_b_, 0.007 ppm), [C3322] (*P*_b_, 0.007 ppm), [F23] (*P*_c_, 0.007 ppm), [U123] (*P*_d_, 0.007 ppm), and [U222] (*P*_d_, 0.007 ppm). The *C*_fs_ > 0 (*P*_a_–*P*_d_) of TPA–BZ DG1 were possibly affected by the structural compositions of [T2̲3] (*P*_a_, 0.029 ppm), [C332̲2] (*P*_b_, 0.037 ppm), [UD] (*P*_d_, 0.029 ppm), and [U2̲33] (*P*_d_, 0.029 ppm).

**Fig. 5:**
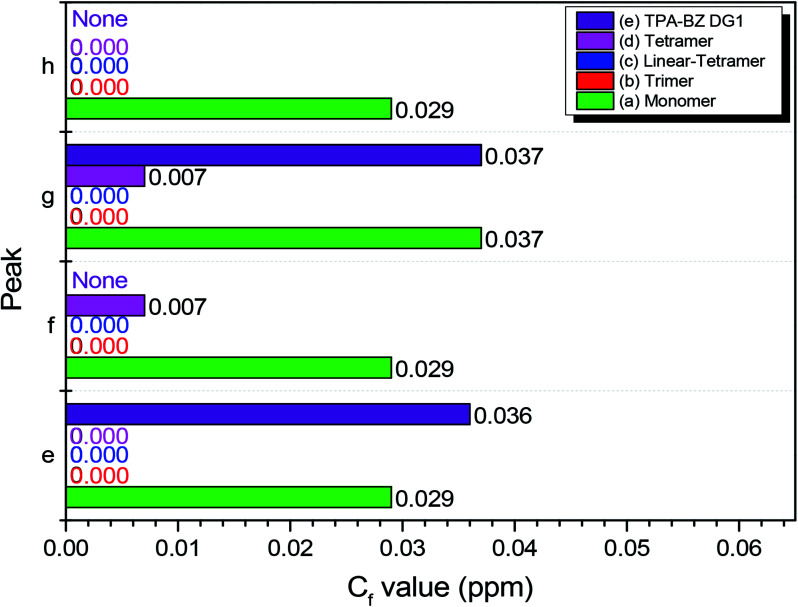
Values of *C*_f_ (peaks e–h) in ^13^C NMR spectra of hyperbranched TPA–BZs and TPA–BZ DG1: (a) monomer, (b) trimer, (c) linear-tetramer, (d) tetramer, and (e) TPA–BZ DG1.

Fig. 5 presents the values of *C*_f_ of P_e_–P_h_ for hyperbranched TPA–BZs and TPA–BZ DG1. The values of *C*_f_ of TPA–BZ monomer were relatively high, all greater than 0.029 ppm. The values of *C*_f_ for *P*_e_ and *P*_g_ of TPA–BZ DG1 were also relatively high, all greater than 0.036 ppm; the spectrum lacked the characteristic signal splits of *P*_f_ and *P*_h_ because of the symmetrical chemical structure of this dendrimer. For the values of *C*_f_ of TPA–BZ tetramer, those of both *P*_f_ and *P*_g_ were equal to 0.007 ppm and those of both *P*_e_ and *P*_h_ were equal to zero. The values of *C*_f_ of TPA–BZ trimer were all zero because of the linear asymmetrical TPA–BZs. Hence, the values of *C*_f_ of both *P*_f_ and *P*_g_ in hyperbranched TPA–BZs (containing four TPA groups) would increase upon increasing the DB. The values of *C*_f_ of both *P*_e_ and *P*_h_ in hyperbranched TPA–BZs (containing four TPA groups) would remain equal to zero. In addition, the value of *C*_f_ of *P*_g_ would appear to be a good candidate for analyzing the DBs of TPA–BZ monomers from 0 to 100%.

Scheme 3 presents the possible mechanism behind the trends in the signals P_e_–P_h_ for hyperbranched TPA–BZs and TPA–BZ DG1. The characteristic signals of P_e_–P_h_ in the ^13^C NMR spectra belong to the oxazine rings of the TPA–BZ monomers, while *P*_f_ and *P*_h_ represent the characteristic signal splits arising from asymmetrical chemical structures; this behavior is similar to that of the *H*_BZ_ portion in ^1^H NMR spectra. As a result, the signal splits of *P*_f_ and *P*_h_ of the oxazine rings can be assigned (Scheme 3) to the BZ rings in red (belonging to the *H*_BZ_ portion of ^1^H NMR spectra) and the signals of *P*_e_ and *P*_g_ of the oxazine rings to the BZ rings in blue (belonging to the *L*_BZ_ portion of ^1^H NMR spectra). The design strategies of the TPA–BZ chemical constructions (Scheme 2) also can be used to examine the relationship between the values of *C*_f_ (*P*_a_–*P*_h_) and the chemical construction developments of TPA–BZ monomer and TPA–BZ trimer. Scheme 3 and Table 2 present the relationship between the values of *C*_f_ and their chemical construction developments as the number of TPA and BZ groups increased for TPA–BZ monomer and TPA–BZ trimer. The values of *C*_f_ of TPA–BZ DG1 ranged from 0.029 to 0.037 ppm, very close to those of TPA–BZ monomer (>0.029 ppm); it is possible that TPA–BZ DG1 was composed of three monomers developed from their focal group directions, resulting in their relative higher values of *C*_f_. The linear TPA–BZ trimer was developed from TPA–BZ monomer through its terminal group directions and had values of *C*_f_ that remained at zero. The branching TPA–BZ tetramer was developed from TPA–BZ trimer through its focal group direction and had values of *C*_f_ ranging from 0 to 0.007 ppm, slightly greater than those of TPA–BZ trimer (*C*_fs_ = 0). linear TPA–BZ tetramer was developed from TPA–BZ trimer through its terminal group directions; it had constant values of *C*_f_ of zero because of its linear TPA–BZ monomers. Hence, the developed directions of TPA–BZ monomer and TPA–BZ trimer strongly affected the values of *C*_f_ of the resulting compounds. Interestingly, the values of *C*_f_ could increase when developing through the direction of the focal group (*e.g.*, TPA–BZ DG1 and TPA–BZ tetramer) or could decrease when developing through the direction of the terminal groups (*e.g.*, trimer and linear-tetramer) of TPA–BZ monomer and TPA–BZ trimer. For the TPA–BZ monomers containing four TPA groups, the values of *C*_f_ increased upon increasing the DB (Table 2); for example, they were zero for linear TPA–BZ tetramer (DB = 0%), from 0 to 0.007 ppm for TPA–BZ tetramer (DB = 75%), and from 0.029 to 0.037 ppm for TPA–BZ DG1 (DB = 100%). Hence, the values of *C*_f_ could also be used to determine the chemical constructions and conformations of the TPA–BZs. Interestingly, the linear asymmetrical TPA–BZs (trimer and linear-tetramer) could not be distinguished in terms of the values of *C*_f_ in their ^13^C NMR spectra; instead, these linear monomers could be identified from their ^1^H NMR spectra (through *H*_BZ_ analysis or the chemical shifts of the signals of the oxazine ring).

### DSC and FTIR spectroscopic analyses of hyperbranched TPA–BZs and TPA–BZ DG1

**Fig. 6:**
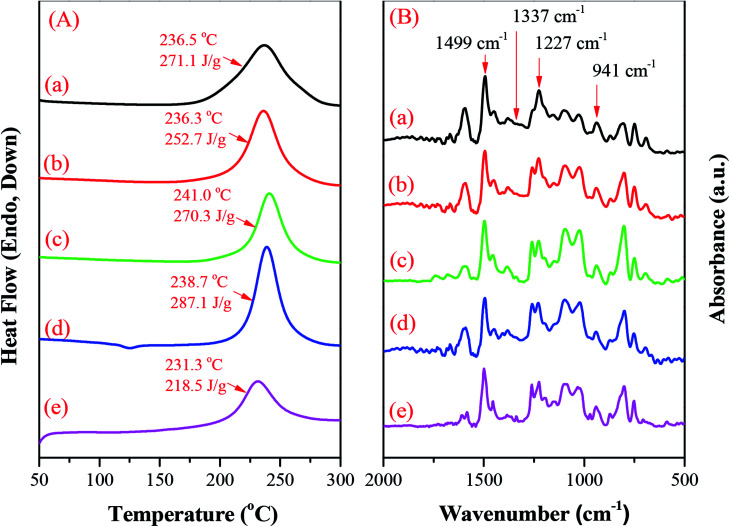
(A) DSC traces and (B) FTIR spectra (recorded at room temperature) of uncured hyperbranched TPA–BZs and TPA–BZ DG1: (a) monomer, (b) trimer, (c) linear-tetramer, (d) tetramer, and (e) TPA–BZ DG1.

We employed DSC to study the thermal behavior of the hyperbranched TPA–BZs and TPA–BZ DG1. Fig. 6A presents the DSC traces (first run) of the uncured hyperbranched TPA–BZs and TPA–BZ DG1. The exothermic peaks of TPA–BZ monomer, TPA–BZ trimer, linear TPA–BZ tetramer, TPA–BZ tetramer, and TPA–BZ DG1 appeared at temperatures of 236.5, 236.3, 241.0, 238.7, and 231.3 °C, respectively—that is, they increased upon increasing the number of TPA units in the hyperbranched TPA–BZs. Interestingly, the temperatures of the exothermic peaks for the TPA–BZ containing four TPA groups decreased upon increasing the DB. For example, the temperature of exothermic peaks followed the trend TPA–BZ DG1 (DB = 100%) < TPA–BZ tetramer (DB = 75%) < linear TPA–BZ tetramer (DB = 0%), indicating that a symmetrical chemical construction led to the exothermic peak appearing at a lower temperature. All four hyperbranched TPA–BZs provided a sharp and symmetrical exothermic peak centered in the range 236–241 °C—lower than that of the conventional Pa-type BZ monomer (3-phenyl-3,4-dihydro-2*H*-benzoxazine, 263 °C)^54^ and the TPA derivatives featuring mono- or di-functional BZ moieties (248–270 °C),^55–57^ implying that these hyperbranched TPA–BZs all have thermal ring-opening polymerization ability and high purity, and significantly improve upon the high temperatures commonly found for ring-opening polymerization of BZ monomers. Interestingly, the temperatures of the exothermic peaks for the hyperbranched TPA–BZs remained higher than those of TPA–BZ dendrimers (231–235 °C).^41^ The temperature of the exothermic peak of the linear symmetrical BZ monomer was, however, higher than that of its asymmetrical counterpart, presumably because a bulky intermediate introduced into the backbone of a symmetrical BZ monomer can move the exothermic peak to higher temperature;^45^ this behavior very different from that of TPA–BZ monomers, possibly due to the branching chemical constructions of TPA–BZs and the incorporation of TPA groups into the monomers.

Fig. 6B presents FTIR spectra of the hyperbranched TPA–BZs and TPA–BZ DG1, recorded at room temperature. The characteristic adsorption peaks of the BZ moieties for these four types of hyperbranched TPA–BZs were located at 1227 (asymmetric COC stretching), 1337 (CH_2_ wagging), 941 and 1499 (vibrations of trisubstituted benzene ring) cm^−1^, confirming the successful incorporation of the BZ groups into hyperbranched TPA–BZs. The characteristic adsorption peaks of the BZ moieties for TPA–BZ DG1 were also located near 1225, 1338, 941 and 1499 cm^−1^. Fig. S5† provides an enlarged view (850 to 1000 cm^−1^) of the FTIR spectra of Fig. 6B. The absorption peak at 941 cm^−1^ has a shoulder peak at 928 cm^−1^, resulting from the asymmetrical chemical construction of the hyperbranched TPA–BZs.^44^ Interestingly, the spectrum of the symmetrical TPA–BZ DG1 also has a shoulder peak at 928 cm^−1^, similar to that of its asymmetrical counterparts—possibly resulting from peak splitting being affected only by the small region of the chemical structure arising from the asymmetrical dendrons of TPA–BZ DG1.

### UV-Vis absorption and PL emission spectra of hyperbranched TPA–BZs and TPA–BZ DG1

**Fig. 7:**
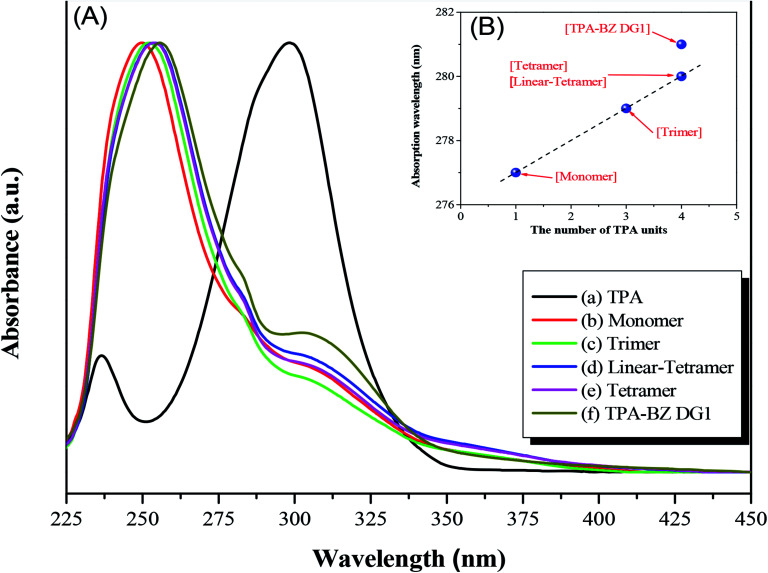
(A) UV-Vis absorbance spectra and (B) absorption wavelengths plotted with respect to the number of TPA units for pristine TPA, hyperbranched TPA–BZs, and TPA–BZ DG1 as solutions in THF (concentration: 10^−4^ M).

We recorded UV-Vis absorption and PL emission spectra to investigate the photophysical properties of the hyperbranched TPA–BZs and TPA–BZ DG1 (Table S5†). Fig. 7A presents the UV-Vis absorption spectra for pure TPA, hyperbranched TPA–BZs and TPA–BZ DG1 in THF at a concentration of 10^−4^ M. Fig. 7A-(a) reveals the characteristic absorption peak centered at 299 nm for pristine TPA, ascribed to the locally excited (LE) π–π* transition. Fig. 7A-(b) reveals three characteristic absorption peaks at 250, 277, and 304 nm for TPA–BZ monomer. We attribute the signal at 250 nm (peak 1) to the high-energy π–π* transition, and those at 277 and 304 nm (peaks 2 and 3, respectively) to the LE π–π* transition and the intramolecular charge-transfer (ICT) π–π* transition, respectively, from the relatively strongly electron donating TPA moieties to the relatively weakly electron donating BZ moieties, as a hybridized local and charge-transfer (HLCT) transition.^58,59^ Upon increasing the number of TPA groups in the hyperbranched TPA–BZs, a small bathochromic shift occurred in the absorption spectrum (peak 2) in Fig. 7A, possibly the result of the increased conjugation length.^60^ Interestingly, the bathochromic shifts of peak 2 for linear TPA–BZ tetramer and TPA–BZ tetramer [Fig. 7A-(d) and (e)] were similar (signals at 280 nm), indicating that the effect of the number of TPA groups was stronger than that of the conformations (DBs of linear TPA–BZ tetramer and TPA–BZ tetramer were 0 and 75%, respectively). The bathochromic shift of peak 2 for TPA–BZ DG1 [Fig. 7A-(f)] was greater than those of linear TPA–BZ tetramer and TPA–BZ tetramer; these three BZ monomers have the same chemical formula (C_126_H_105_N_13_O_9_), but different DBs, especially in the symmetrical TPA–BZ dendrimers, resulting in a significant increase in the conjugation length. Prasad *et al.* reported that as the branching number of the TPA derivatives increased, bathochromic shifts were observed in UV–Vis absorption and one-photon emission spectra, resulting in an enhancement in the TPA cross section,^42^ due to an enhancement in vibronic coupling, on the basis of a theoretical study.^61^ In addition, we also observed a bathochromic shift for peak 1 that was similar to that of peak 2. Fig. 7B presents the absorption wavelength in the UV-Vis spectra of the hyperbranched TPA–BZs and TPA–BZ DG1 plotted with respect to the number of TPA groups. The absorption wavelengths of these hyperbranched TPA–BZs were linearly related to the number of TPA groups, even for the different conformations of the monomers of linear TPA–BZ tetramer and TPA–BZ tetramer. TPA–BZ DG1 exhibited an absorption wavelength relatively higher than those of linear TPA–BZ tetramer and TPA–BZ tetramer, due to the significantly greater conjugation length of the TPA–BZ dendrimer (DB = 100%).^42^

**Fig. 8:**
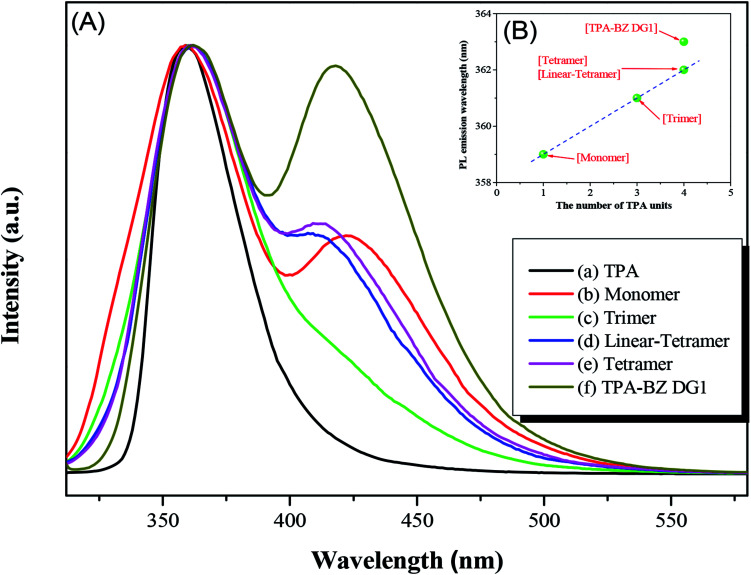
(A) PL (excitation wavelength: 300 nm) spectra and (B) PL emission wavelengths plotted with respect to the number of TPA units for pristine TPA, hyperbranched TPA–BZs, and TPA–BZ DG1 as solutions in THF (concentration: 10^−4^ M).

Fig. 8A presents the PL emission spectra for pure TPA, hyperbranched TPA–BZs, and TPA–BZ DG1 in THF at a concentration of 10^−4^ M after excitation at a wavelength of 300 nm. Fig. 8A-(a) reveals that the emission peak was centered at 359 nm for pristine TPA, the result of the LE π–π* transition.

Fig. 8A-(b) reveals two PL emission peaks for TPA–BZ monomer at 359 and 422 nm (peaks L and H, respectively), possibly the results of an HLCT transition and an aggregation occurred, respectively. Upon increasing the number of TPA groups in the hyperbranched TPA–BZs, peak L in the PL emission spectrum shifted from 359 to 362 nm for both TPA–BZ monomer and TPA–BZ tetramer; this small bathochromic shift possibly resulted from the increase in the conjugation length.^60^ Interestingly, the bathochromic shifts of peak L for linear TPA–BZ tetramer and TPA–BZ tetramer were similar (signals at 362 nm), suggesting that the effect of the number of TPA groups was stronger than that of the conformations; this phenomenon is similar to that observed in the UV-Vis spectra. In addition, peak H for TPA–BZ trimer had intensity lower than those of the other hyperbranched TPA–BZs, implying that TPA–BZ trimer was dispersed well in THF and less likely than the other TPA–BZ monomers to aggregate.

The bathochromic shift of peak L for TPA–BZ DG1 [Fig. 8A-(f)] was greater than those of linear TPA–BZ tetramer and TPA–BZ tetramer, similar to the behavior in the UV-Vis spectra and possibly due to the DB increasing, especially for the symmetrical TPA–BZ dendrimers, resulting in greater conjugation lengths.^42,61^ The intensity of peak H for TPA–BZ DG1 was higher than that of TPA–BZ tetramer because the symmetrical dendrimer aggregated more readily than did its asymmetrical counterpart. Fig. 8B presents the PL emission wavelengths of the hyperbranched TPA–BZs and TPA–BZ DG1 plotted with respect to the number of TPA groups. The PL emission wavelengths of these hyperbranched TPA–BZs corresponded linearly with the number of TPA groups, even for the different conformation monomers for linear TPA–BZ tetramer and TPA–BZ tetramer. TPA–BZ DG1 exhibited a PL emission wavelength higher than those of linear TPA–BZ tetramer and TPA–BZ tetramer, due to the greater conjugation length of the TA–BZ dendrimer.^42^ The UV-Vis absorption and PL emission spectra of the hyperbranched TPA–BZs featured small bathochromic shifts that were affected strongly by the number of TPA groups, presumably because of the increase in the effective conjugation length.^41–43^ Interestingly, the effective conjugation length of the TPA–BZs was not affected by the DB of the asymmetrical hyperbranched monomers, except in the case of the symmetrical dendrimers; for example, TPA–BZ DG1 possesses an effective conjugation length relatively higher than those of the asymmetrical hyperbranched TPA–BZs (*e.g.*, TPA–BZ trimer and linear TPA–BZ tetramer) because the branching number increased significantly for the TPA–BZ dendrimers.

### Thermal ring-opening polymerization of hyperbranched TPA–BZs and TPA–BZ DG1

**Fig. 9:**
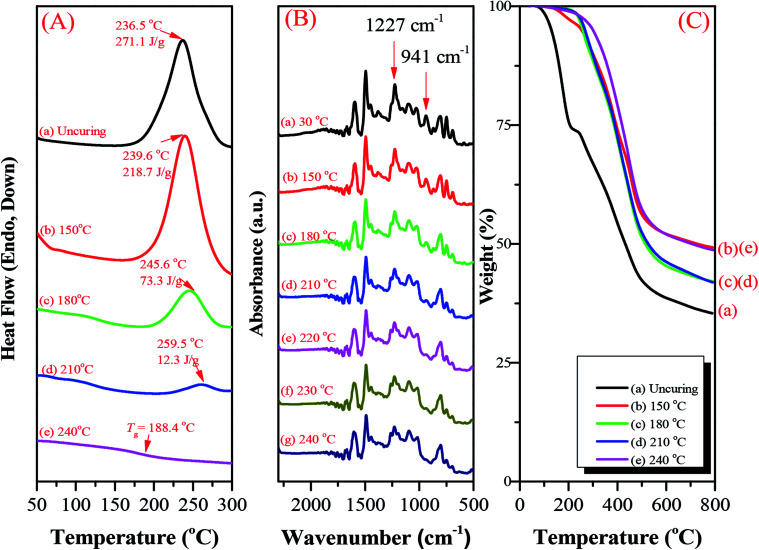
(A) DSC traces, (B) FTIR spectra, and (C) TGA analysis before and after curing of TPA–BZ monomer at various temperatures, recorded after each curing stage.

The hyperbranched TPA–BZs and TPA–BZ DG1 possess the ability to undergo thermal ring-opening polymerization. Scheme S6† presents the possible chemical structure of the polymerized TPA–BZ tetramer. We employed DSC, FTIR spectroscopy, and TGA to study the thermal ring-opening polymerization of the hyperbranched TPA–BZs and TPA–BZ DG1. DSC revealed the thermal polymerization behavior of TPA–BZ monomer before and after curing at 150, 180, 210, and 240 °C (Fig. 9A). Fig. 9A-a presents the DSC trace of the uncured monomer; a sharp exothermic peak was centered at 236.5 °C with a reaction heat of 271.1 J g^−1^. The reaction heat of the exothermic peak decreased gradually upon increasing the curing temperature, eventually disappearing at 240 °C, implying that the full curing state of the polybenzoxazine had been achieved. The temperature of the exothermic peak increased upon increasing the curing temperature, presumably because the relatively higher crosslinking density of the monomer inhibited its thermal curing ability.^35^Fig. 9A-e reveals a value of *T*_g_ of 188.4 °C after curing at 240 °C, revealing an enhancement in the crosslinking density and suggesting that the full curing state of the polybenzoxazine had been achieved at 240 °C. FTIR spectroscopy revealed the thermal polymerization behavior of TPA–BZ monomer before and after curing at 150, 180, 210, 220, 230, and 240 °C (Fig. 9B). The characteristic adsorption peaks of the BZ moieties for TPA–BZ monomer appeared at 1227 and 941 cm^−1^. The characteristic adsorption peak at 941 cm^−1^ (out of plane C–H bending) disappeared completely from the spectrum of TPA–BZ monomer after curing at 240 °C (Fig. 9B-g), suggesting that the full curing stage of BZ moieties had been achieved; this behavior is consistent with the results from the DSC analysis. Hence, we selected a curing temperature of 240 °C for our subsequent studies.

We used TGA to examine the thermal polymerization behavior of TPA–BZ monomer. Fig. 9C presents its TGA thermograms before and after curing at 150, 180, 210, and 240 °C under a N_2_ atmosphere; the values of *T*_d_ of TPA–BZ monomer before and after curing at these temperatures were 129.4, 253.2, 257.4, 267.2, and 299.9 °C, respectively, with char yields of 35.4, 49.3, 42.1 41.9, and 48.7 wt%, respectively. Thus, the values of *T*_d_ and the char yields both increased upon increasing the thermal curing temperature, resulting in an increased crosslink density. Hence, TPA–BZ monomer features higher thermal stability and higher char yield comparing to the conventional Pa-type BZ monomer.^54^

**Fig. 10:**
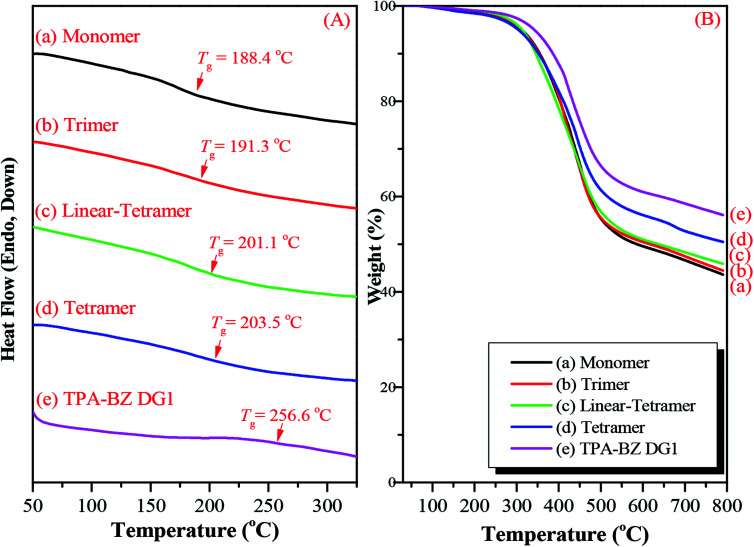
(A) DSC traces and (B) TGA analysis of hyperbranched TPA–BZs and TPA–BZ DG1 after curing at 240 °C: (a) monomer, (b) trimer, (c) linear-tetramer, (d) tetramer, and (e) TPA–BZ DG1, recorded after each curing stage.

We also employed DSC and TGA to study the thermal ring-opening polymerization of the hyperbranched TPA–BZs and TPA–BZ DG1. We used DSC to study the thermal behavior of the hyperbranched TPA–BZs and TPA–BZ DG1 after curing at 240 °C, recorded at a heating rate of 20 °C min^−1^. Fig. 10A presents the DSC traces for the hyperbranched TPA–BZs and TPA–BZ DG1 with curing at 240 °C, recorded after each curing stage. The values of *T*_g_ of TPA–BZ monomer, TPA–BZ trimer, linear TPA–BZ tetramer, TPA–BZ tetramer, and TPA–BZ DG1 after curing at 240 °C were 188.4, 191.3, 201.1, 203.5, and 256.6 °C, respectively. The value of *T*_g_ of TPA–BZ DG1 was higher than those of the other hyperbranched monomers because the segmental mobility in the polymer network was restricted by its dendrimer core group.^45^ We used TGA to investigate the thermal stability of the hyperbranched TPA–BZs and TPA–BZ DG1 after curing at 240 °C (Fig. 10B). The values of *T*_d_ and the char yields for TPA–BZ monomer, TPA–BZ trimer, linear TPA–BZ tetramer, TPA–BZ tetramer, and TPA–BZ DG1 were 311.3, 311.9, 313.0, 304.1, and 342.0 °C, respectively, and 43.6, 44.5, 45.9, 50.5, and 56.1 wt%, respectively. The thermal stability of TPA–BZ DG1 was greater than those of the other hyperbranched monomers, again because of its symmetrical structure.

## Conclusions

We have prepared a series of well-defined thermal polymerizable hyperbranched polymers through facile one-pot Mannich condensations using a unique feeding approach. The signals representing the oxazine rings in NMR spectra revealed a tendency to shift downfield as the DB of the TPA–BZs increased, except in the case of the ^13^C NMR spectra of the linear asymmetrical monomers. In addition, we used the structural compositions as a powerful tool to investigate the *H*_BZ_/*L*_BZ_ portions and values of *C*_f_ for the TPA–BZ monomers. The temperature of the exothermic peak for the thermal polymerizations of the TPA–BZ monomers decreased as the DB increased, such that it was lower than those of conventional BZ monomers; accordingly, this method overcomes the common problem of curing temperatures of BZ monomers being too high. UV-Vis absorption and PL emission spectra revealed bathochromic shifts of the hyperbranched TPA–BZs that increased as the number of TPA groups increased, presumably because of the greater effective conjugation length; these shifts for TPA–BZ tetramer and linear TPA–BZ tetramer were smaller than that of the symmetrical dendrimer counterpart TPA–BZ DG1, possibly because of the significant increase in effective conjugation length of the dendrimer caused by the greater branching number. DSC and FTIR spectroscopy revealed that these hyperbranched TPA–BZs underwent full curing at 240 °C to form polybenzoxazines. The polymerized TPA–BZ DG1 displayed thermal properties, in terms of values of *T*_g_ and *T*_d_, superior to those of the hyperbranched TPA–BZ polybenzoxazines because the segmental mobility in the polymer network was restricted by the dendrimer core group and because of the symmetrical construction.

## Conflicts of interest

There are no conflicts to declare.

## Supplementary Material

RA-008-C8RA00506K-s001

## References

[cit1] Kim Y. H. (1998). J. Polym. Sci., Part A: Polym. Chem..

[cit2] Gao C., Yan D. (2004). Prog. Polym. Sci..

[cit3] Voit B. I., Lederer A. (2009). Chem. Rev..

[cit4] Tomalia D. A., Naylor A. M., Goddard W. A. (1990). Angew. Chem., Int. Ed..

[cit5] Hawker C. J., Fréchet J. M. J. (1990). J. Am. Chem. Soc..

[cit6] Voit B. (2000). J. Polym. Sci., Part A: Polym. Chem..

[cit7] Wilms D., Stiriba S. E., Frey H. (2010). Acc. Chem. Res..

[cit8] Yates C. R., Hayes W. (2004). Eur. Polym. J..

[cit9] Maier G., Zech C., Voit B., Komber H. (1998). Macromol. Chem. Phys..

[cit10] Smet M., Schacht E., Dehaen W. (2002). Angew. Chem., Int. Ed..

[cit11] Sinananwanich W., Ueda M. (2008). J. Polym. Sci., Part A: Polym. Chem..

[cit12] Huang W. G., Su L. J., Bo Z. S. (2009). J. Am. Chem. Soc..

[cit13] Liu N., Vignolle J., Vincent J. M., Robert F., Landais Y., Cramail H., Taton D. (2014). Macromolecules.

[cit14] Ohta Y., Fujii S., Yokoyama A., Furuyama T., Uchiyama M., Yokozawa T. (2009). Angew. Chem., Int. Ed..

[cit15] Ohta Y., Kamijyo Y., Fujii S., Yokoyama A., Yokozawa T. (2011). Macromolecules.

[cit16] Yang D., Kong J. (2016). Polym. Chem..

[cit17] Guan Z. B., Cotts P. M., McCord E. F., McLain S. J. (1999). Science.

[cit18] Guan Z. (2002). J. Am. Chem. Soc..

[cit19] Hong C. Y., You Y. Z., Wu D. C., Liu Y., Pan C. Y. (2007). J. Am. Chem. Soc..

[cit20] Segawa Y., Higashihara T., Ueda M. (2010). J. Am. Chem. Soc..

[cit21] Suzuki M., Ii A., Saegusa T. (1992). Macromolecules.

[cit22] Suzuki M., Yoshida S., Shiraga K., Saegusa T. (1998). Macromolecules.

[cit23] Sunder A., Hanselmann R., Frey H., Mulhaupt R. (1999). Macromolecules.

[cit24] Bharathi P., Moore J. S. (2000). Macromolecules.

[cit25] Möck A., Burgath A., Hanselmann R., Frey H. (2001). Macromolecules.

[cit26] Kainthan R. K., Muliawan E. B., Hatzikiriakos S. G., Brooks D. E. (2006). Macromolecules.

[cit27] Bharathi P., Moore J. S. (1997). J. Am. Chem. Soc..

[cit28] Bernal D. P., Bedrossian L., Collins K., Fossum E. (2003). Macromolecules.

[cit29] Segawa Y., Higashihara T., Ueda M. (2013). Polym. Chem..

[cit30] Ohashi S., Kilbane J., Heyl T., Ishida H. (2015). Macromolecules.

[cit31] Zhang W. F., Froimowicz P., Arza C. R., Ohashi S., Xin Z., Ishida H. (2016). Macromolecules.

[cit32] Ghosh N. N., Kiskan B., Yagci Y. (2007). Prog. Polym. Sci..

[cit33] Mohamed M. G. , Lin R. C., Hsu K. C., Zhang W., Zhong X. and Kuo S. W., Mediated surface properties of polybenzoxazines. in Advanced and emerging polybenzoxazine science and technology, ed. H. Ishida and P. Froimowicz, Elsevier, Amsterdam, 2017, ch. 12, pp. 205–219

[cit34] Mohamed M. G. , Lin R. C. and Kuo S. W., Polybenzoxazine/carbon nanotube composites. in Advanced and emerging polybenzoxazine science and technology, ed. H. Ishida and P. Froimowicz, Elsevier, Amsterdam, 2017, ch. 36, pp. 725–738

[cit35] Lin R. C., Mohamed M. G., Hsu K. C., Wu J. Y., Jheng Y. R., Kuo S. W. (2016). RSC Adv..

[cit36] Lin R. C., Mohamed M. G., Chen T., Kuo S. W. (2017). Polymers.

[cit37] Mohamed M. G., Kuo S. W. (2016). Polymers.

[cit38] Kim H. M., Cho B. R. (2009). Chem. Commun..

[cit39] Ning Z. J., Tian H. (2009). Chem. Commun..

[cit40] Pawlicki M., Collins H. A., Denning R. G., Anderson H. L. (2009). Angew. Chem., Int. Ed..

[cit41] Lin R. C., Mohamed M. G., Kuo S. W. (2017). Macromol. Rapid Commun..

[cit42] Chung S. J., Kim K. S., Lin T. H., He G. S., Swiatkiewicz J., Prasad P. N. (1999). J. Phys. Chem. B.

[cit43] Paul G. K., Mwaura J., Argun A. A., Taranekar P., Reynolds J. R. (2006). Macromolecules.

[cit44] Yang P., Gu Y. (2012). J. Appl. Polym. Sci..

[cit45] Sini N. K., Endo T. (2016). Macromolecules.

[cit46] Kolanadiyil S. N., Minami M., Endo T. (2017). Macromolecules.

[cit47] Hawker C. J., Lee R., Fréchet J. M. J. (1991). J. Am. Chem. Soc..

[cit48] Hölter D., Burgath A., Frey H. (1997). Acta Polym..

[cit49] Deng Y. Y., Zhang Q., Zhang H. C., Zhang C. X., Wang W. H., Gu Y. (2014). Ind. Eng. Chem. Res..

[cit50] Zhang Q., Yang P., Deng Y. Y., Zhang C. X., Zhu R. Q., Gu Y. (2015). RSC Adv..

[cit51] Hakala K., Nuutinen J. M. J., Straub T., Rissanen K., Vainiotalo P. (2002). Rapid Commun. Mass Spectrom..

[cit52] Nuñez-Dallos N., Reyes A., Quevedo R. (2012). Tetrahedron Lett..

[cit53] Zhang L., Zhu Y. J., Li D., Wang M., Chen H. B., Wu J. S. (2015). RSC Adv..

[cit54] Ishida H. , Overview and historical background of polybenzoxazine research. in Handbook of benzoxazine resins, ed. H. Ishida and T. Agag, Elsevier, Amsterdam, 2011, ch. 1, pp. 3–81

[cit55] Lin L. C., Yen H. J., Kung Y. R., Leu C. M., Lee T. M., Liou G. S. (2014). J. Mater. Chem. C.

[cit56] Lin L. C., Yen H. J., Chen C. J., Tsai C. L., Liou G. S. (2014). Chem. Commun..

[cit57] Shih H. K., Chu Y. L., Chang F. C., Zhu C. Y., Kuo S. W. (2015). Polym. Chem..

[cit58] Wu J. H., Chen W. C., Liou G. S. (2016). Polym. Chem..

[cit59] Lin C. H., Shih Y. S., Wang M. W., Tseng C. Y., Juang T. Y., Wang C. F. (2014). RSC Adv..

[cit60] Yan Z. Q., Xu B., Dong Y. J., Tian W. J., Li A. W. (2011). Dyes Pigm..

[cit61] Macak P., Luo Y., Norman P., Ågren H. (2000). J. Chem. Phys..

