# Supplementary material for: Well-defined benzoxazine/triphenylamine-based hyperbranched polymers with controlled degree of branching
Source: RSC Adv. 2018 Apr 11;8(24):13592–611. doi: 10.1039/c8ra00506k (PMC9079809; doi:10.1039/c8ra00506k)
Supplement: RA-008-C8RA00506K-s001 [file RA-008-C8RA00506K-s001.pdf]

Electronic Supplementary Material (ESI) for RSC Advances.
This journal is © The Royal Society of Chemistry 2018

Supporting Information for

Well-Defined Benzoxazine/Triphenylamine-Based Hyperbranched Polymers with Controlled Degree of Branching

Ruey-Chorng Lin, and Shiao-Wei Kuo*

Department of Materials and Optoelectronic Science, National Sun Yat-Sen University, Kaohsiung, 804, Taiwan

E-mail: kuosw@faculty.nsysu.edu.tw

Symmetrical analysis of values of C_f (P_a–P_d) in ^{13}C NMR spectra of TPA-BZs

The code rules of the structural compositions of the t-BZs in this study were as follows (Table S4): (i) the arrow in red indicates the position of the designated carbon atom; (ii) the t-BZ composition of [Uxxx]: "U" indicates the t-BZ composition and "xxx" indicates three b-BZ sets connected to the same center nitrogen atom (e.g., [U133] indicates that the t-BZ composition had one focal and two terminal function sets connected to the same center nitrogen atom); (iii) a t-BZ composition of [UD] indicates the core group of the TPA-BZ dendrimer.

The 25 types of structural compositions (including 8 types of t-BZ structural compositions) were used to analyze the values of C_f in the ^{13}C NMR spectra of the hyperbranched TPA-BZs and TPA-BZ DG1 (Table S4). For example, TPA-BZ Trimer is composed of the five types of structural compositions: four pieces of [T23] (P_a), two pieces of [C3312] (P_b), two pieces of [F22] (P_b and P_c, respectively), one piece of [U122] (P_d), and two pieces of [U233] (P_d). These structural compositions could be used to analyze the relationships between the chemical structures and the values of C_f in the ^{13}C NMR spectra. A systematic approach was employed to analyze the relationship between the values of C_f and the chemical structures of the hyperbranched TPA-BZs and TPA-BZ DG1, using these structural compositions. Accordingly (Table S4), there were 14

types of structural compositions that could possibly lead to values of C_f (P_a–P_d) greater than zero ($C_{fs} > 0$) in ^{13}C NMR spectra, including [T13] (P_a), [T$\underline{2}$3] (P_a), [T12] (P_a), [C33$\underline{2}$2] (P_b), [C2213] (P_b), [C3322] (P_b), [F33] (P_b and P_c), [F23] (P_c), [U133] (P_d), [UD] (P_d), [U$\underline{2}$33] (P_d), [U123] (P_d), and [U222] (P_d). In other words, the chemical construction of TPA-BZ monomers possessing these 14 structural compositions might possibly lead to $C_{fs} > 0$ (P_a–P_d) in ^{13}C NMR spectra; for example, six types of structural compositions of TPA-BZ Tetramer would result in $C_{fs} > 0$ (P_a–P_d): one piece of [T12] (P_a), one piece of [C2213] (P_b), two pieces of [C3322] (P_b), one piece of [F23] (P_c), one piece of [U123] (P_d), and one piece of [U222] (P_d). The values of C_f (Table S4) are consistent with the calculated results from the ^{13}C NMR spectra (Table 1). These structural compositions can be used to determine the relationship between the values of C_f in ^{13}C NMR spectra and the chemical structures of the hyperbranched TPA-BZs and TPA-BZ dendrimers.

Table S1. The analysis of H_{BZ} and L_{BZ} in ^{1}H NMR spectra of TPA-BZs.

Item	Structural composition	Code	Mono	Trimer	L-Tetra	Tetra	DG1	Note
1	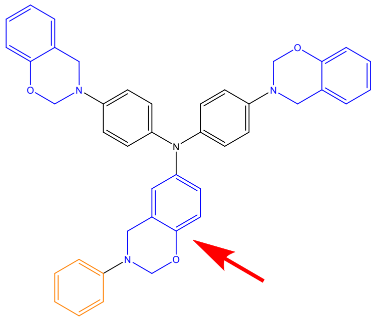	[F33]	1					H_{BZ}
2	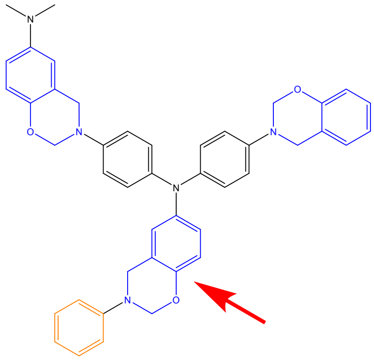	[F23]				1		H_{BZ}
3	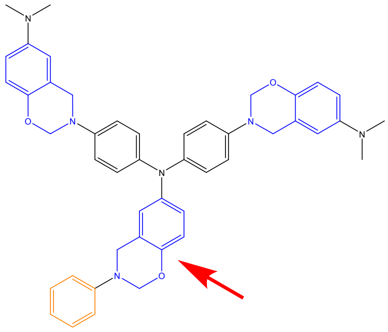	[F22]		1	1			H_{BZ}
4	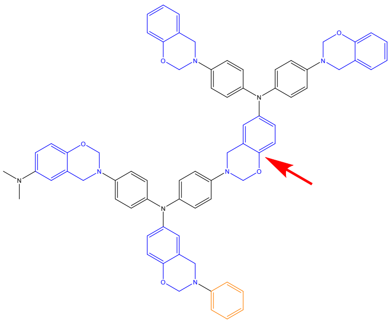	[C3312]		2	1			H_{BZ}

5	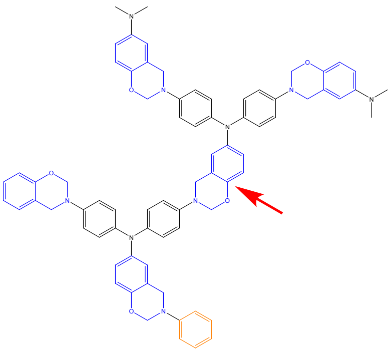	[C2213]				1		H_{BZ}
6	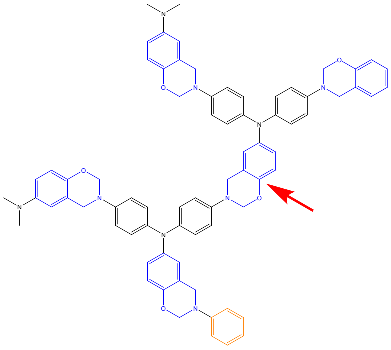	[C2312]			1			L_{BZ}
7	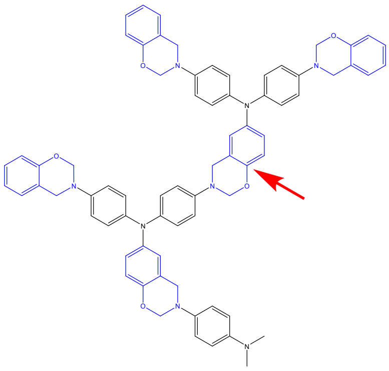	[C3323]			1			L_{BZ}
8	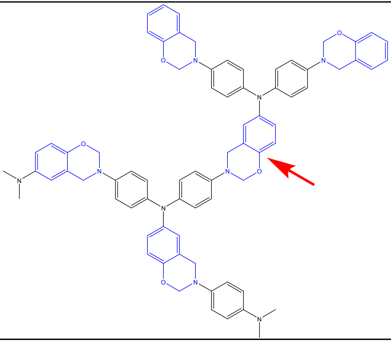	[C3322]				2		L_{BZ}

9	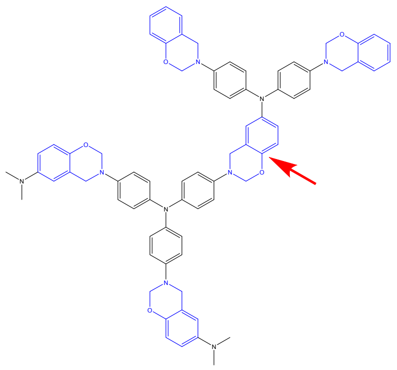	[C3322]					3	L_{BZ}
10	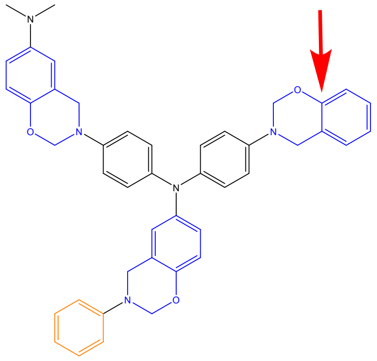	[T12]				1		H_{BZ}
11	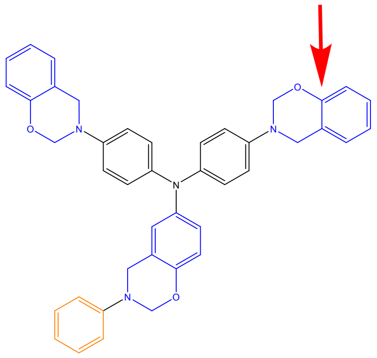	[T13]	2					L_{BZ}
12	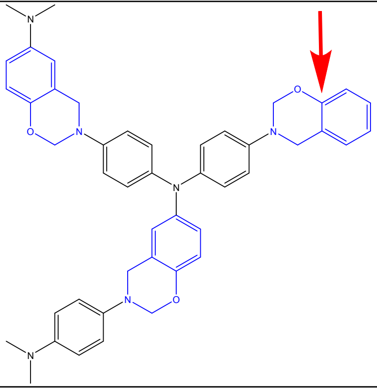	[T22]			1			L_{BZ}

13	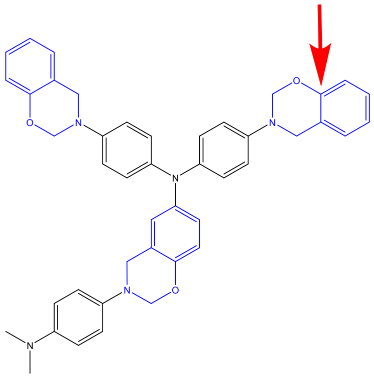	[T23]		4	4	4		L_{BZ}
14	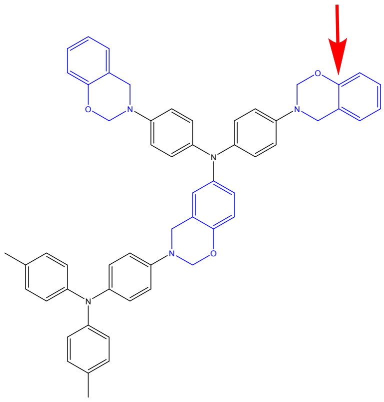	[T$\underline{2}$3]					6	L_{BZ}
H_{BZ}			1	3	2	3	0	
L_{BZ}			2	4	7	6	9	
Overall BZ groups			3	7	9	9	9	

Notice: 1. Sample names of “Mono”, “L-Tetra”, “Tetra”, and “DG1” indicate “Monomer”, “Linear-Tetramer”, “Tetramer”, and “TPA-BZ DG1”, respectively.

2. The code rules of the structural compositions (composed by several b-BZ sets and nitrogen atoms) in this study are presented as following: (i) the arrow in red indicates the position of the designated BZ ring (^{1}H NMR spectra); (ii) the numbers 1, 2, $\underline{2}$, and 3 represent the b-BZ sets serving the focal function, the connection function, the connection function featuring a connected component of a core group of the TPA-BZ dendrimer, and the terminal function, respectively; (iii) the focal composition of [Fxx]: “F” indicates the focal composition and “xx” indicates that the b-BZ set of the focal function has two b-BZ sets at its end nitrogen atom close to the BZ ring (e.g., [F33] indicates that one focal function set has two terminal function sets at its end nitrogen atom closed to the BZ ring); (iv) the connection composition of [Cxxyy]: “C” indicates the connection composition, “xx” indicates that the b-BZ set of the connection function has two b-BZ sets at its end nitrogen atom close to the BZ ring, and “yy” indicates that the b-BZ set of the connection function has two b-BZ sets at its end nitrogen atom close

to the benzyl group (e.g., [C3312] indicates that one connection function set has two terminal function sets at its end nitrogen atom close to the BZ ring, and one focal and one connection function set at its end nitrogen atom close to the benzyl group); (v) the terminal composition of [Txx]: "T" indicates the terminal composition and "xx" indicates that the b-BZ set of the terminal function has two b-BZ sets at its end nitrogen atom close to the benzyl group (e.g., [T12] indicates that one terminal function set has one focal and one connection function set at its end nitrogen atom close to the benzyl group).

Table S2. H_{BZ} analysis for compounds developed in directions from the focal group and terminal groups.

Developed direction		From the focal group		From the terminal groups	
Original compound ↓ Developed compound		Monomer ↓ TPA-BZ DG1	Trimer ↓ Tetramer	Monomer ↓ Trimer	Trimer ↓ Linear-Tetramer
H_{BZ}	Original	1	3	1	3
	Developed	0	1	3	2

Notice: (a) The developed directions of the compound refer to the Scheme 2.
(b) The H_{BZ} values of "Original" and "Developed" indicate that the H_{BZ} values of the original compound before and after developed compound formation, respectively.

Table S3. Chemical shifts of peaks a–h in ^{13}C NMR spectra of TPA-BZs.

Peak	a	b	c	d	e	f	g	h
Monomer	153.963	151.135	147.816	143.633	79.212	78.662	49.401	48.895
Trimer	153.905	151.106	147.787	143.604	79.183	78.633	49.364	48.866
Linear-Tetramer	153.905	151.106	147.787	143.604	79.183	78.633	49.364	48.866
Tetramer	153.912	151.113	147.794	143.611	79.183	78.640	49.371	48.866
TPA-BZ DG1	153.934	151.143	—	143.633	79.219	—	49.401	—

Table S4. The analysis of C_f (P_a–P_d) in ^{13}C NMR spectra of TPA-BZs.

Item	Structural composition	Code	Peak	Mono	Trimer	L-Tetra	Tetra	DG1
1	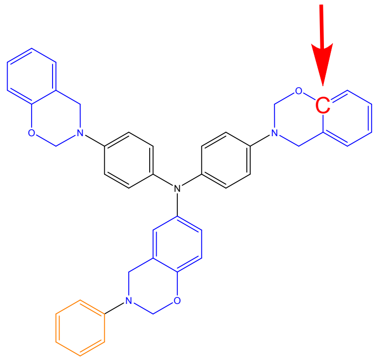	[T13]	a	2 (0.058)				
2	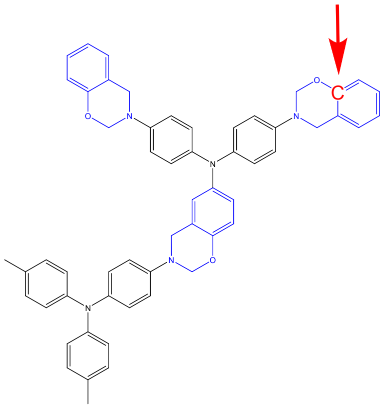	[T23]	a					6 (0.029)
3	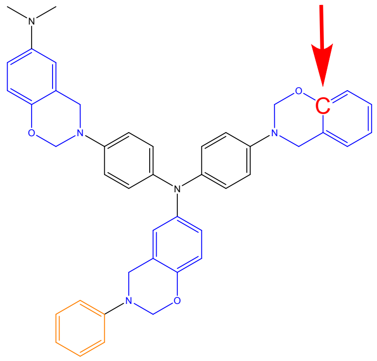	[T12]	a				1 (0.007)	

4	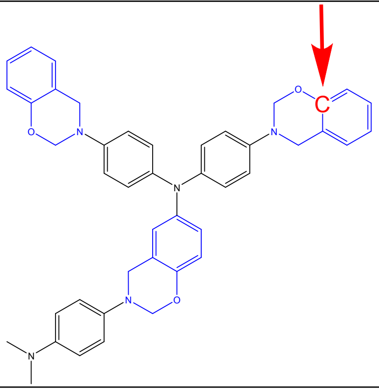	[T23]	a		4	4	4	
5	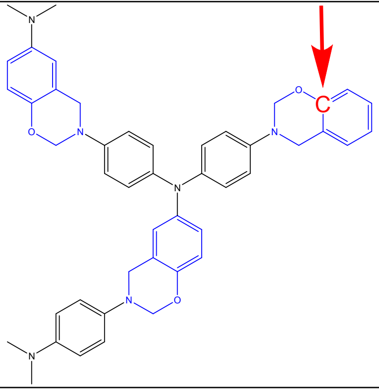	[T22]	a			1		
6	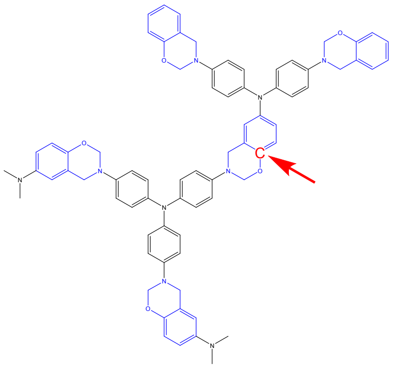	[C3322]	b					3 (0.037)
7	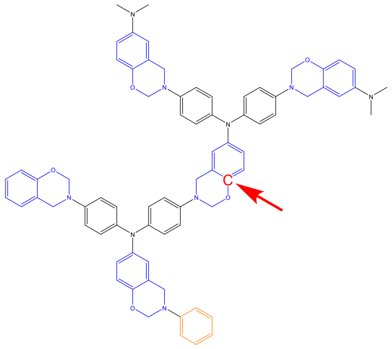	[C2213]	b				1 (0.007)	

8	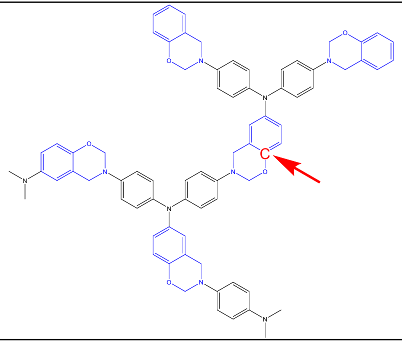	[C3322]	b				2<br>(0.007)	
9	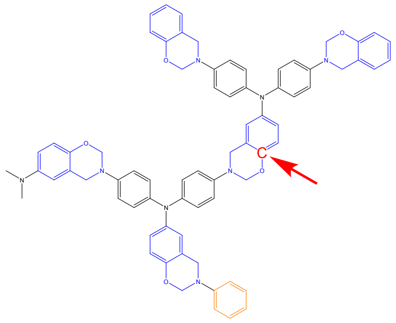	[C3312]	b		2	1		
10	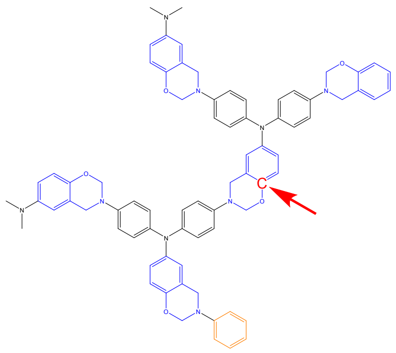	[C2312]	b			1		
11	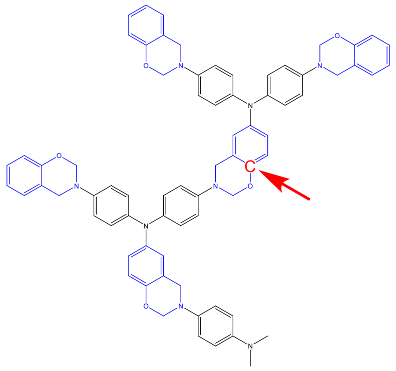	[C3323]	b			1		
12	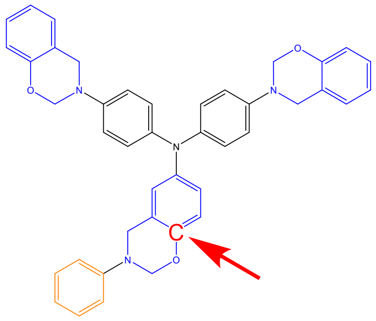	[F33]	b	1<br>(0.029)				

13	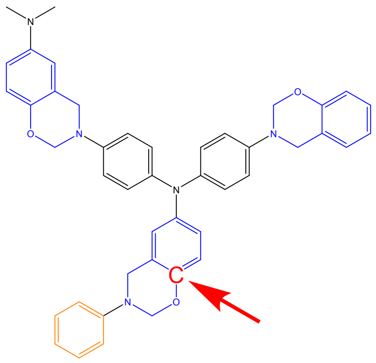	[F23]	b				1	
14	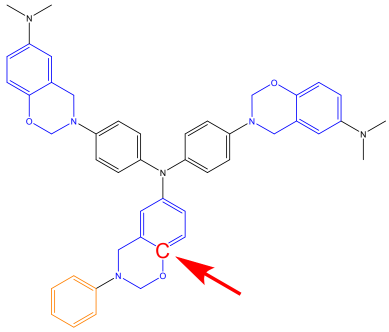	[F22]	b		1	1		
15	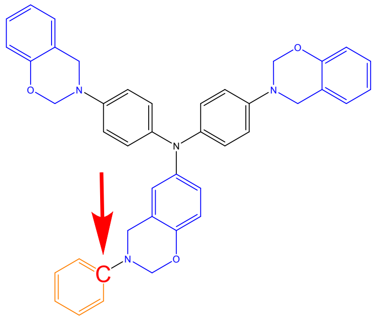	[F33]	c	1 (0.029)				
16	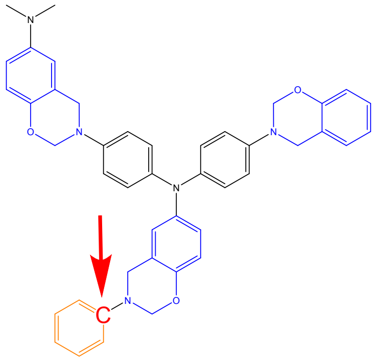	[F23]	c				1 (0.007)	
17	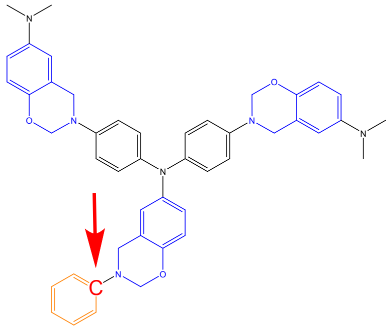	[F22]	c		1	1		

18	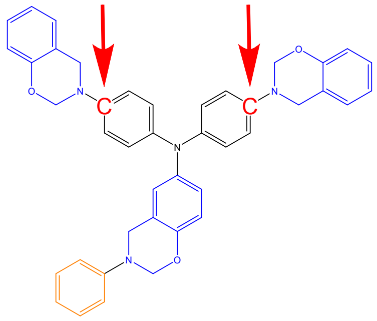	[U133]	d	1 (0.029)				
19	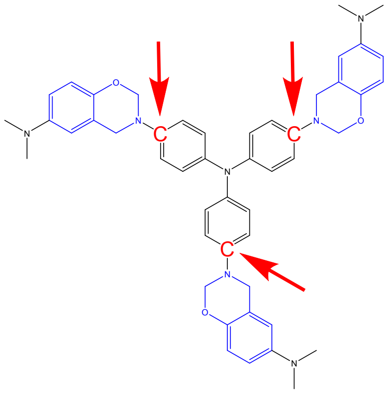	[UD]	d					1 (0.029)
20	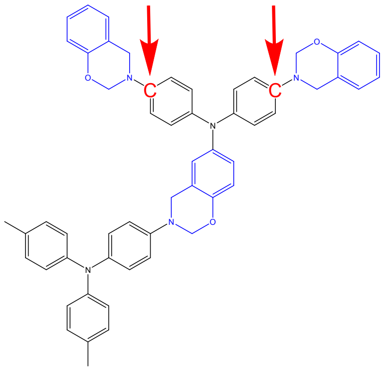	[U233]	d					3 (0.029)
21	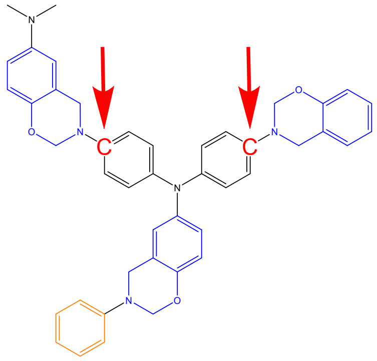	[U123]	d				1 (0.007)	

22	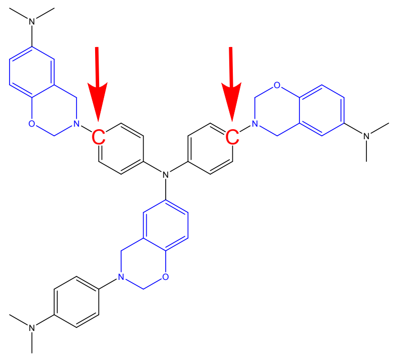	[U222]	d				1<br>(0.007)	
23	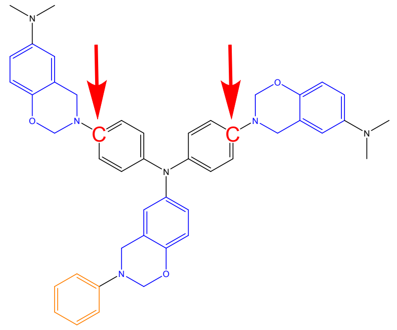	[U122]	d		1	1		
24	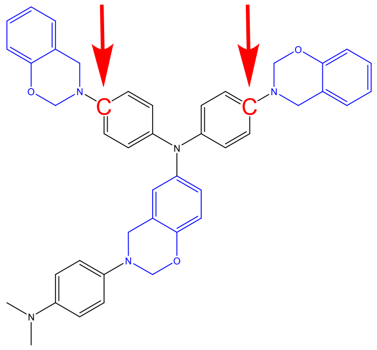	[U233]	d		2	2	2	
25	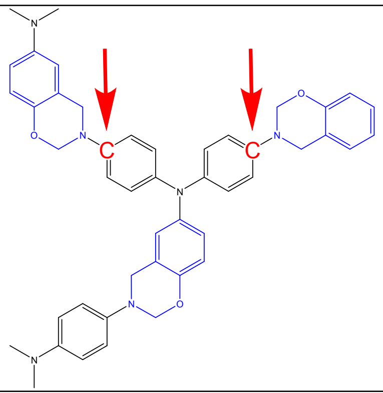	[U223]	d			1		

Notice: 1. Sample names of "Mono", "L-Tetra", "Tetra", and "DG1" indicate "Monomer", "Linear-Tetramer", "Tetramer", and "TPA-BZ DG1", respectively.

2. The C_f values (ppm) are indicated in table with a symbol of bracket, except for the C_f = 0.

3. The code rules of the structural compositions (composed by several b-BZ sets and nitrogen atoms, or a t-BZ composition) in this study are presented as following: (i) the arrow in red indicates the position of the designated carbon atom (^{13}C NMR spectra);

(ii) the numbers 1, 2, $\underline{2}$, and 3 represent the b-BZ sets serving the focal function, the connection function, the connection function featuring a connected component of a core group of the TPA-BZ dendrimer, and the terminal function, respectively; (iii) the focal composition of [Fxx]: “F” indicates the focal composition and “xx” indicates that the b-BZ set of the focal function has two b-BZ sets at its end nitrogen atom close to the BZ ring (e.g., [F33] indicates that one focal function set has two terminal function sets at its end nitrogen atom closed to the BZ ring); (iv) the connection composition of [Cxxyy]: “C” indicates the connection composition, “xx” indicates that the b-BZ set of the connection function has two b-BZ sets at its end nitrogen atom close to the BZ ring, and “yy” indicates that the b-BZ set of the connection function has two b-BZ sets at its end nitrogen atom close to the benzyl group (e.g., [C3312] indicates that one connection function set has two terminal function sets at its end nitrogen atom close to the BZ ring, and one focal and one connection function set at its end nitrogen atom close to the benzyl group); (v) the terminal composition of [Txx]: “T” indicates the terminal composition and “xx” indicates that the b-BZ set of the terminal function has two b-BZ sets at its end nitrogen atom close to the benzyl group (e.g., [T12] indicates that one terminal function set has one focal and one connection function set at its end nitrogen atom close to the benzyl group); (vi) the t-BZ composition of [Uxxx]: “U” indicates the t-BZ composition and “xxx” indicates three b-BZ sets connected to the same center nitrogen atom (e.g., [U133] indicates that the t-BZ composition had one focal and two terminal function sets connected to the same center nitrogen atom); (vii) a t-BZ composition of [UD] indicates the core group of the TPA-BZ dendrimer.

Table S5. Photophysical properties of pristine TPA and TPA-BZs.

Sample	UV-Vis absorption spectra		PL emission spectra	
	λ_{max}^{abs}	I_{max}^{abs}	λ_{max}^{em}	I_{max}^{em}
TPA	299	1.000	359	1.000
Monomer	250	1.000	359	1.000
	277	0.411	422	0.556
	304	0.247	—	—
Trimer	252	1.000	361	1.000
	279	0.406	414	0.316
	304	0.216	—	—
Linear-Tetramer	254	1.000	362	1.000
	280	0.439	409	0.561
	304	0.269	—	—
Tetramer	254	1.000	362	1.000
	280	0.432	412	0.583
	304	0.250	—	—
TPA-BZ DG1	255	1.000	363	1.000
	281	0.475	418	0.952
	301	0.324	—	—

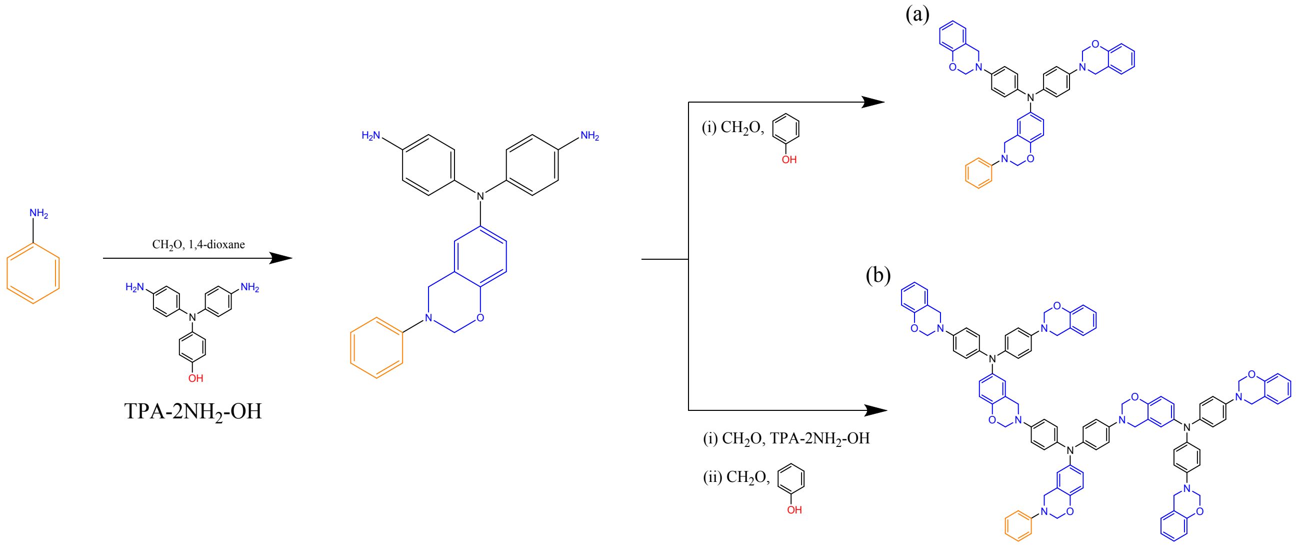

Scheme S1. Synthesis of hyperbranched TPA-BZs: (a) Monomer and (b) Trimer.

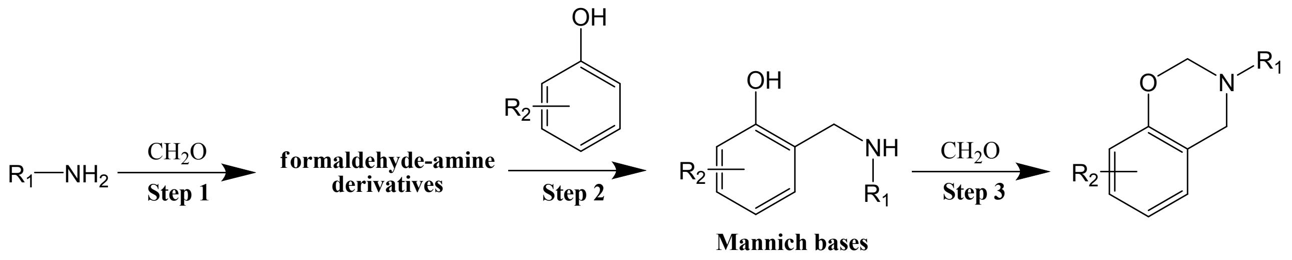

Scheme S2. Mechanism of Mannich reaction forming BZ from a phenol, a primary amine, and formaldehyde.

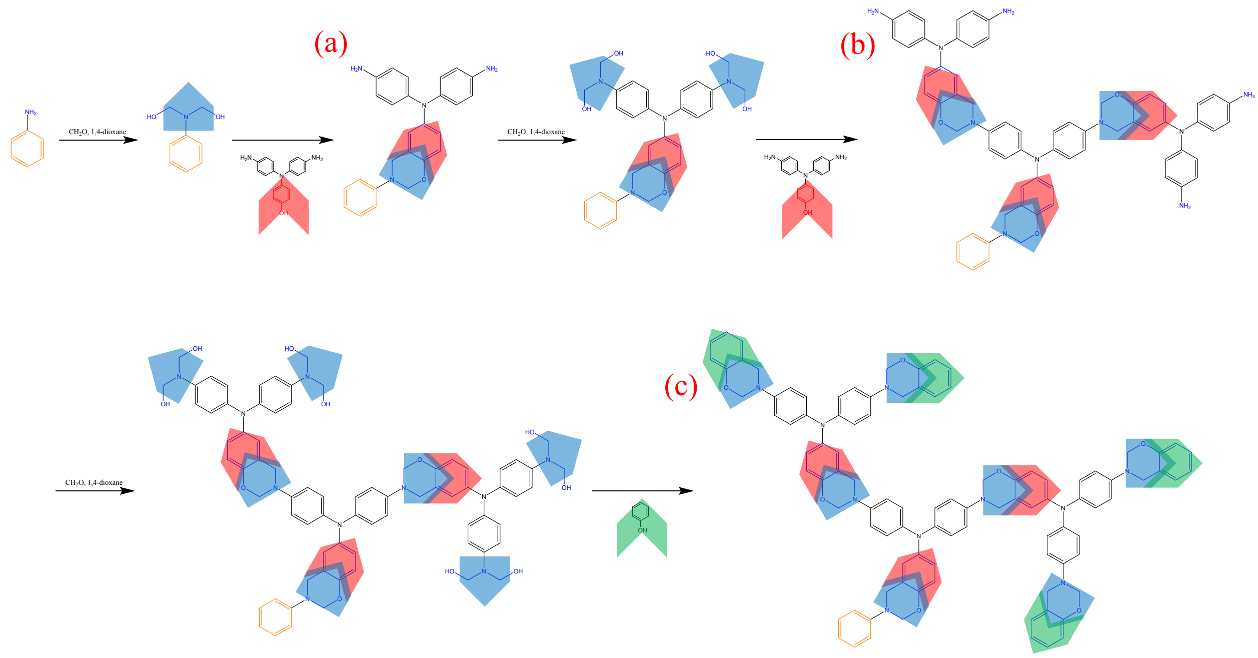

Scheme S3. Possible mechanism for the preparation of Trimer.

(A)

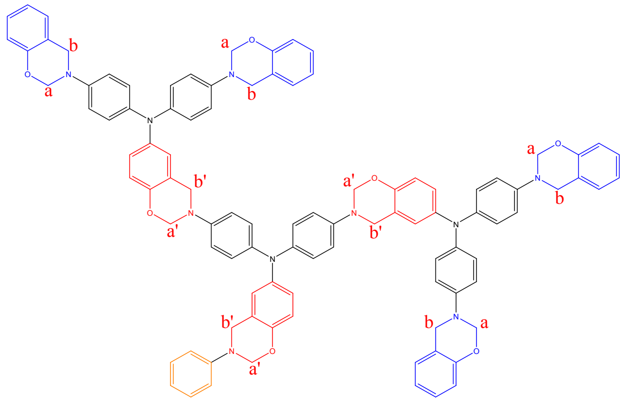

(B)

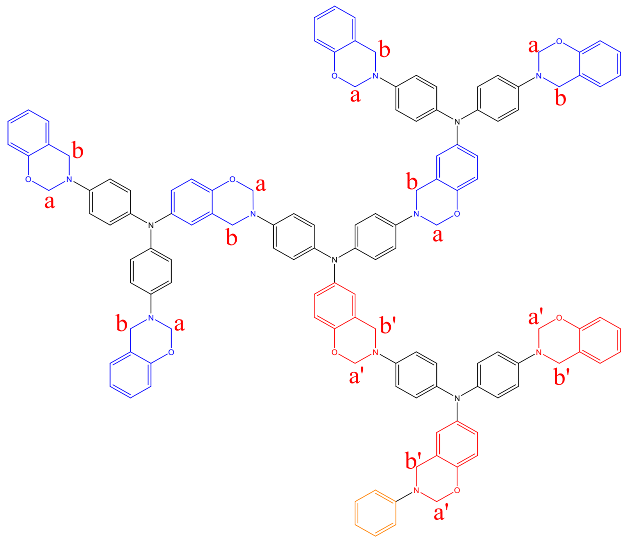

Scheme S4. Chemical structures of ^{1}H NMR spectra of (A) Trimer and (B) Tetramer.

(A)

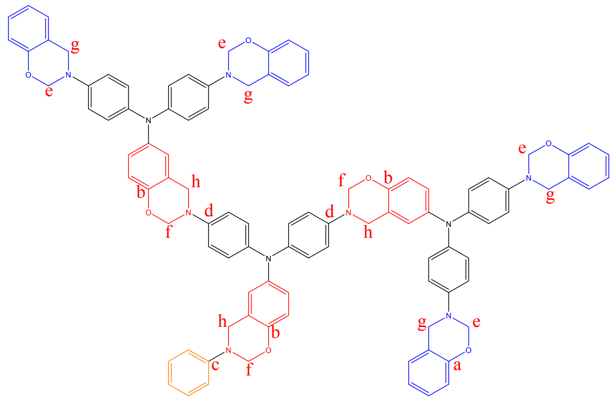

(B)

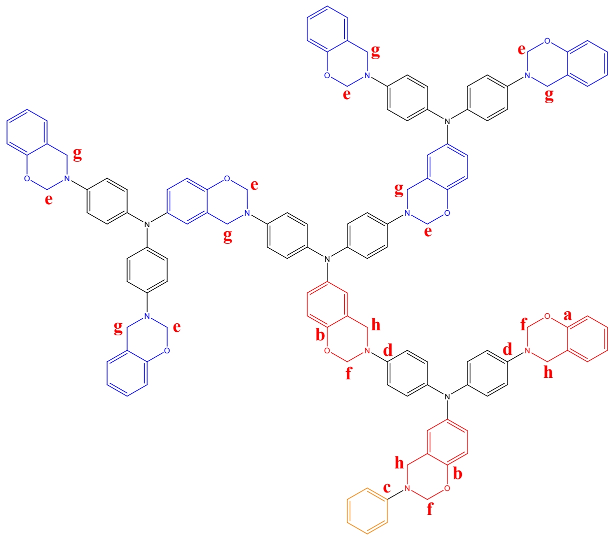

Scheme S5. Chemical structures of ^{13}C NMR spectra of (A) Trimer and (B) Tetramer.

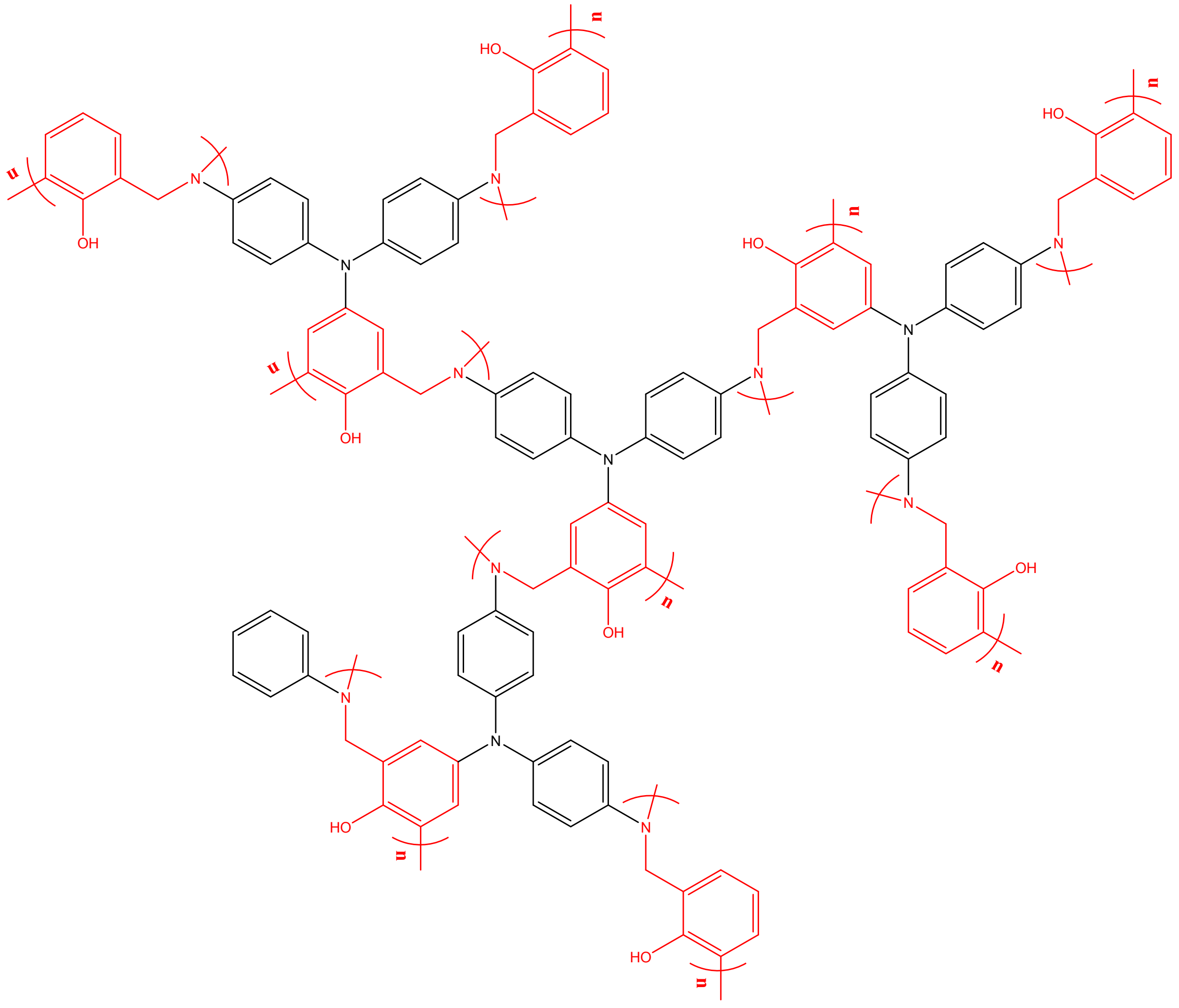

Scheme S6. Possible chemical structure of Tetramer after thermal ring-opening polymerization.

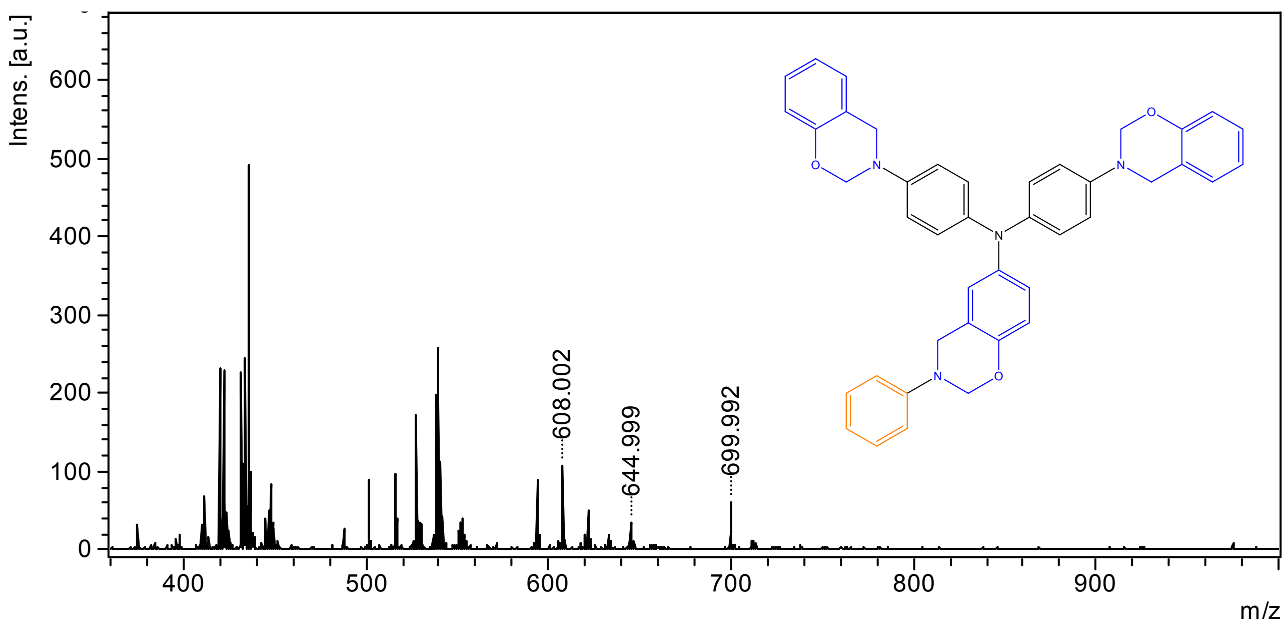

Figure S1. MALDI-TOF mass spectrum of Monomer.

^{13}C NMR TPA-BZ Hyperbranched Monomer

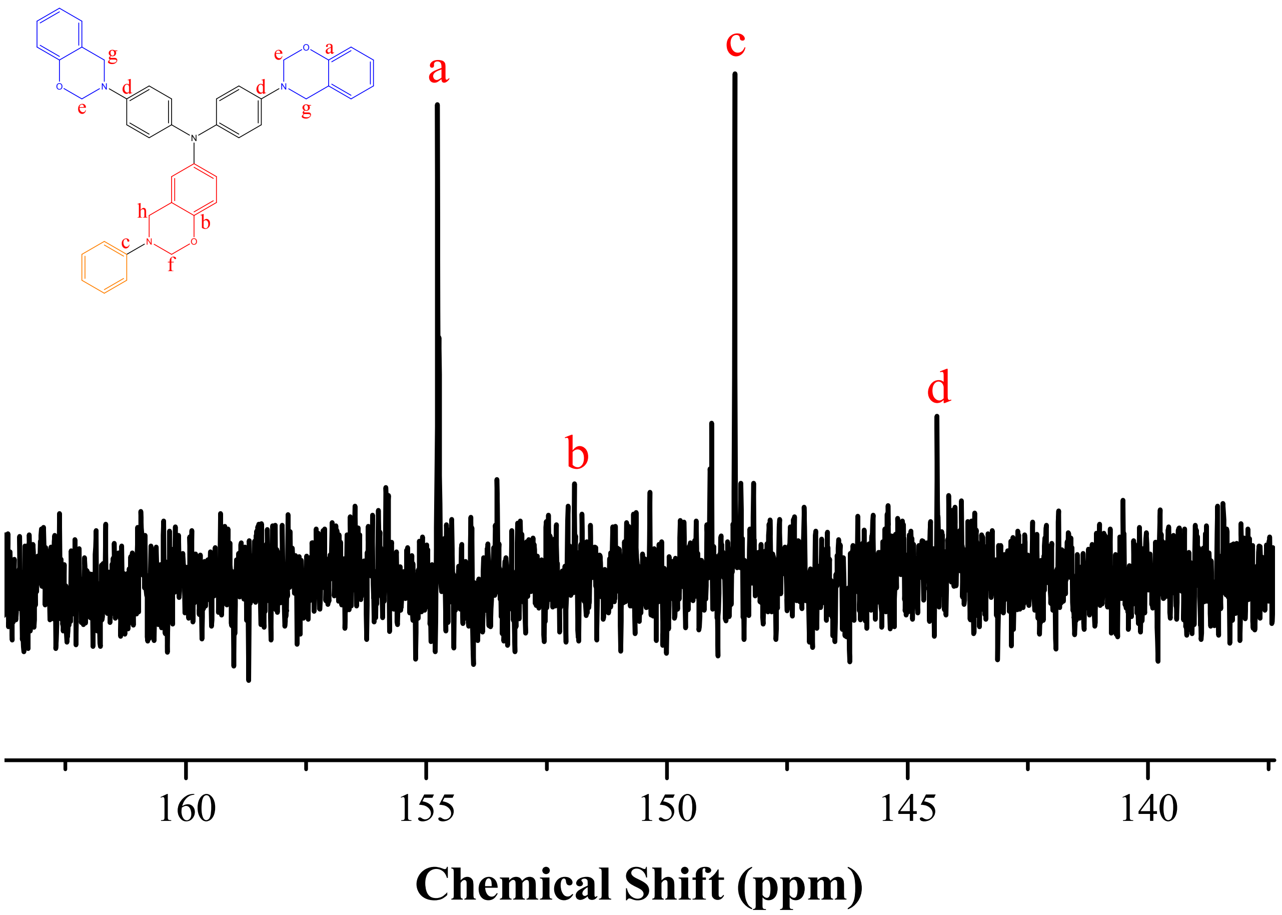

Figure S2. Enlarged view (from 138 to 168 ppm) of ^{13}C NMR spectrum of Monomer.

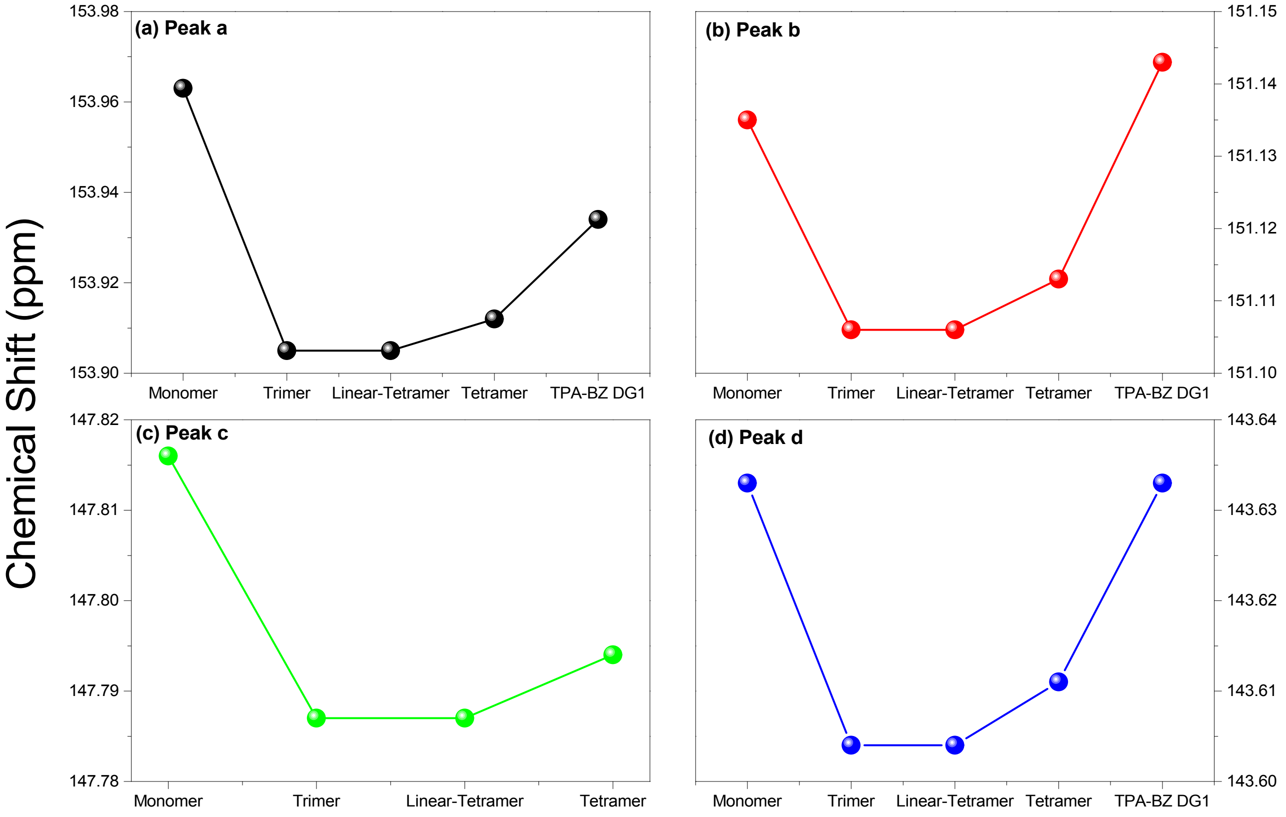

Figure S3. Chemical shifts in ^{13}C NMR spectra of hyperbranched TPA-BZs and TPA-BZ DG1: Peaks (a) a, (b) b, (c) c, and (d) d.

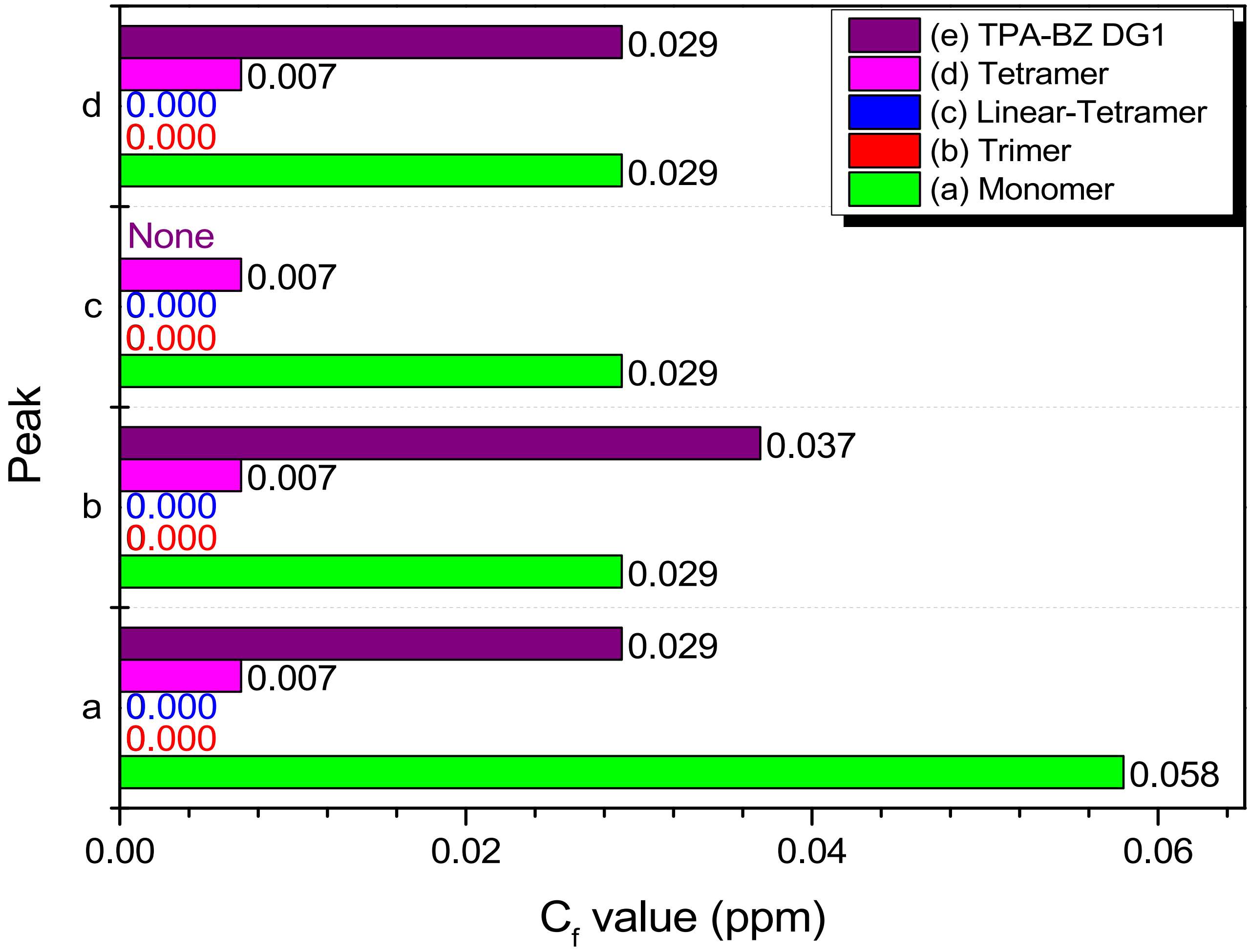

Figure S4. Values of C_f (peaks a–d) in ^{13}C NMR spectra of hyperbranched TPA-BZs and TPA-BZ DG1: (a) Monomer, (b) Trimer, (c) Linear-Tetramer, (d) Tetramer, and (e) TPA-BZ DG1.

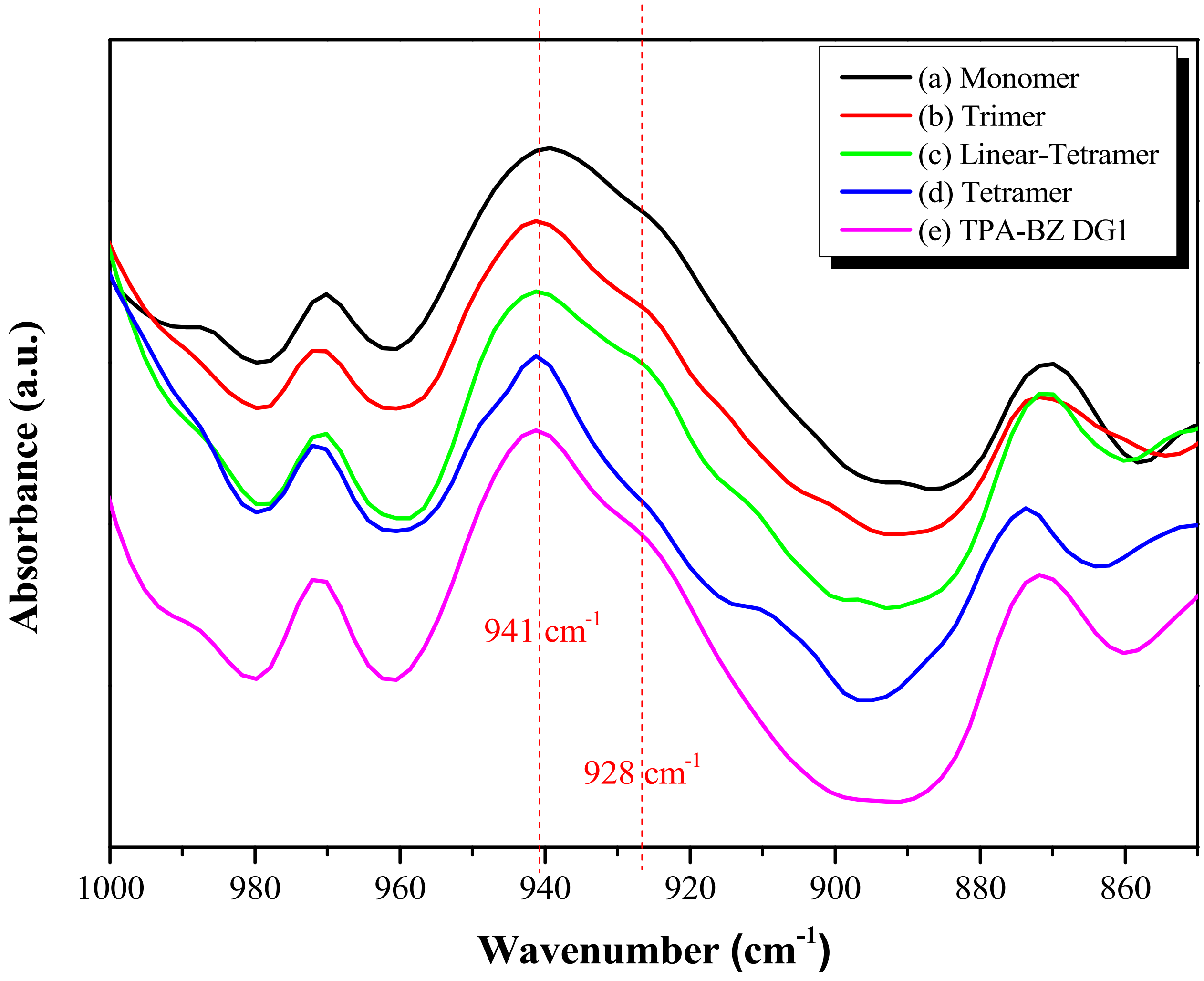

Figure S5. FTIR spectra of TPA-BZs, in the range 850–1000 cm^{-1}, recorded at room temperature: (a) Monomer, (b) Trimer, (c) Linear-Tetramer, (d) Tetramer, and (e) TPA-BZ DG1.
